# Rationalizing Counterion Selection for the Development of Lipophilic Salts: A Case Study with Venetoclax

**DOI:** 10.1021/acs.molpharmaceut.4c00106

**Published:** 2024-05-04

**Authors:** Callum D. Ryan, Brendan T. Griffin, Joseph P. O’Shea

**Affiliations:** † School of Pharmacy, University College Cork, College Road, Cork T12 K8AF, Ireland; ‡ SSPC, the Science Foundation Ireland Research Centre for Pharmaceuticals, School of Pharmacy, University College Cork, Cork T12 K8AF, Ireland

**Keywords:** lipophilic salt, lipid-based formulation, venetoclax, poorly water-soluble drug, drug absorption

## Abstract

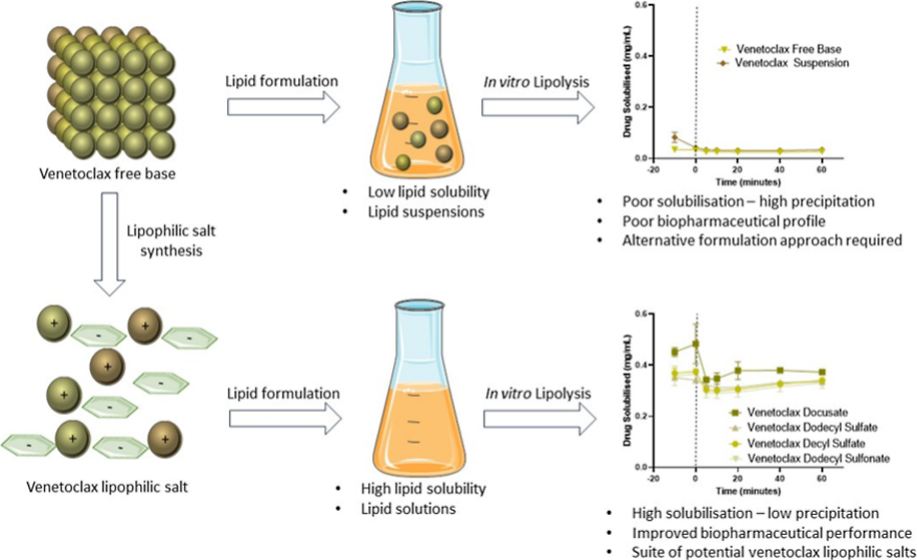

The use of lipid-based formulations (LBFs) can be hindered by low dose loading due to solubility limitations of candidate drugs in lipid vehicles. Formation of lipophilic salts through pairing these drugs with a lipophilic counterion has been demonstrated as a potential means to enhance dose loading in LBFs. This study investigated the screening of appropriate counterions to form lipophilic salts of the BCS class IV drug venetoclax. The physical properties, lipid solubility, and *in vitro* performance of the salts were analyzed. This study illustrated the versatility of alkyl sulfates and sulfonates as suitable counterions in lipophilic salt synthesis with up to ∼9-fold higher solubility in medium- and long-chain LBFs when compared to that of the free base form of venetoclax. All salts formulated as LBFs displayed superior *in vitro* performance when compared to the free base form of the drug due to the higher initial drug loadings in LBFs and increased affinity for colloidal species. Further, *in vitro* studies confirmed that venetoclax lipophilic salt forms using alkyl chain counterions demonstrated comparable *in vitro* performance to venetoclax docusate, thus reducing the potential for laxative effects related to docusate administration. High levels of the initial dose loading of venetoclax lipophilic salts were retained in a molecularly dispersed state during dispersion and digestion of the formulation, while also demonstrating increased levels of saturation in biorelevant media. The findings of this study suggest that alkyl chain sulfates and sulfonates can act as a suitable alternative counterion to docusate, facilitating the selection of counterions that can unlock the potential to formulate venetoclax as an LBF.

## 1 Introduction

Despite the success of lipid-based formulations (LBFs) as bioenabling formulations for poorly water-soluble drugs,^1,2^ their utility remains underexploited, with falling proportions of LBFs among newly licensed drug products.^3^ One possible reason for this underutilization is inadequate lipid solubility of many novel drug candidates, preventing adequate dosing in LBFs, a process crucial for bypassing the rate-limiting dissolution step.^1,4,5^ Several methods exist to overcome the challenge of limited drug solubility in lipid vehicles. Supersaturation is one such example, whereby a drug is present in lipid excipients at concentrations above the thermodynamic solubility in the formulation, which has been induced through heating the formulation, and this has been extensively reviewed by Holm and colleagues.^6^ The addition of fatty acids to the formulation is another method of boosting drug solubility in lipid excipients by leveraging favorable interactions between the basic drug and fatty acids.^7^ Finally, conversion of free base forms of a drug to lipophilic salts or ionic liquids through metathesis reactions with acidic counterions has been proven as an efficient means of improving drug solubility in lipid excipients.^8−10^

Synthesis of lipophilic salts requires fundamental considerations related to appropriate counterion selection, including capacity to form a stable salt complex, reduction in crystalline energy, an increase in lipophilicity of the salt complex compared to that of the free base, and no toxicological implications. The stability of the complex is reliant on the drug-counterion p*K*_a_ difference, where a Δp*K*_a_ of greater than 2 between the drug and counterion is required to ensure complete proton transfer and formation of a stable salt complex.^11^ Counterions are typically selected for their ability to disrupt molecular packing through stereochemical hindrance. Ideal model counterions should have high molecular weight and steric bulk, lower localized charge density, minimal hydrogen bonding potential, an asymmetrical structure to disrupt crystal packing, fewer heteroatoms, and increased degrees of freedom.^9,12^ This disruption to the molecular packing of the salt complex generally correlates with a decreased melting point of the lipophilic salt due to a reduction in the crystalline solid state forces.^8,13,14^ Lipophilic salts that display a melting point of less than 100 °C are classified as ionic liquids.^15^ Ionic liquids incur the same benefits as typical lipophilic salts but are considered even more favorable due to their lower hydrophobic burden from the disrupted crystal lattice, which may allow for an even higher dose loading in the LBF. Generally, ionic liquids are liquids at room temperature, allowing for favorable solubilization within the lipid excipients.^11,15,16^

Addressing the solubility enhancement of a salt in a lipid vehicle extends beyond merely reducing the crystal lattice energy; it also involves optimizing the intermolecular interactions between the lipophilic salt and the lipid vehicle. Therefore, a suitable counterion should not only disrupt the crystal lattice energy but also exhibit lipophilicity. The interactions between the lipophilic salt and the lipid vehicle must be strong enough to overcome solute–solute interactions, instead promoting solute–solvent interactions, contributing significantly to solubility enhancement.^8,17^ Therefore, an ideal counterion for lipophilic salt synthesis should display both lipophilicity and structural characteristics that are suitable to disrupt the crystal lattice energy and form a stable salt. Finally, it is crucial to consider the toxicity of potential lipophilic salts, and, therefore for the synthesis of lipophilic salts, it is imperative to select counterions that are either listed in the FDA’s “Generally Regarded as Safe” (GRAS) list as of 2023 or have a well-documented history of safe usage.^18^ Numerous drugs have successfully utilized the lipophilic salt/LBF formulation approach to increase the *in vivo* performance of the drug, including venetoclax, lumefantrine, cinnarizine, ceritinib, amlodipine, metformin, and itraconazole.^9,10,12,19,20^ Despite these advancements, a commercial lipophilic salt–LBF product has not yet been introduced to the market.

**Figure 1:**
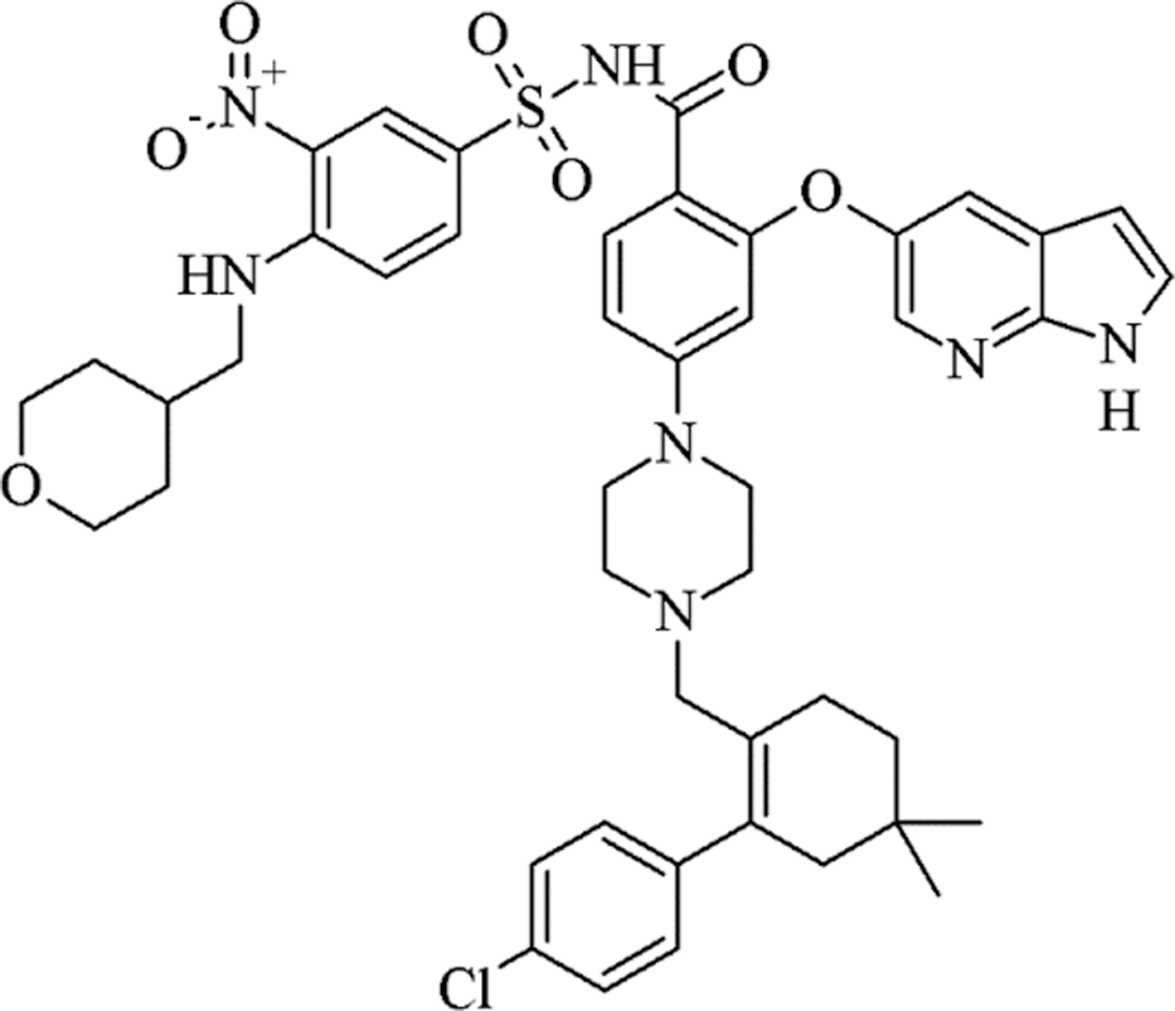
Molecular structure of venetoclax free base.^24^

For this study, the BCS class IV drug venetoclax (Figure 1), which is a selective B-cell lymphoma-2 inhibitor licensed for the treatment of leukemia in 2016, is used as the model compound. Venetoclax was selected due to its poor solubility in both medium- and long-chain triglycerides (<1 mg/mL), limiting its suitability for formulation as an LBF.^5^ The commercial form of venetoclax—Venclyxto—is an amorphous solid dispersion that displays a food effect, with a 3.4-fold increase in oral bioavailability when taken with a low-fat meal and a 5-fold increase after a high-fat meal with respect to venetoclax being taken in the fasted state.^21^ As a result, venetoclax would benefit from the synergistic lipophilic salt and LBF approach to both enhance oral bioavailability and negate potential food effects. Previous work has demonstrated that venetoclax can be synthesized as a lipophilic salt using docusate as a counterion. Other counterions such as oleic acid and decanoic acid were also trialled in the formation of the lipophilic salt of venetoclax; however, they proved to be unsuccessful.^20^ While docusate is GRAS approved, the molecule can exert physiological effects and has been used as a stool softener. Doses of docusate in excess of 100 mg per dose if present as a counterion could potentially lead to unwanted side effects such as diarrhea, if administered chronically.^22^ In an effort to explore alternative counterions, alkyl sulfates and sulfonates are proposed as a viable alternative due to their suitable p*K*_a_ for proton transfer.^11^ The alkyl chain of these molecules has also been proven to sufficiently disrupt molecular packing for other lipophilic salts.^8^ Sulfates and sulfonates also display favorable charge distribution, reducing electrostatic interactions between the anion and cation and consequently leading to reduced melting temperature.^19^ Alkyl sulfates and sulfonates typically display low toxicity and appear in several commercial products.^23^ In addition, with a venetoclax docusate dose that is equivalent to 100 mg of venetoclax, 49 mg of docusate is also administered. By contrast, as the alkyl sulfates/sulfonates all have lower molecular weights, lower doses of counterions are coadministered as part of the salt form; thus, a higher dose loading can be achieved using alkyl sulfates/sulfonates as counterions when compared to that using docusate. The risk of a laxative effect is also reduced through the absence of the docusate if an alkyl sulfate/sulfonate is used.

This study aims to assess the utility of these alkyl chain sulfates and alkyl chain sulfonates as alternative counterions to docusate in the formation of lipophilic salts of venetoclax. Exploring the utility of alkyl chain counterions would allow for a rational selection of counterions to be used in the formation of a lipophilic salt of venetoclax. Venetoclax lipophilic salts using alkyl chain sulfates and sulfonates of varying chain lengths were integrated into both medium- and long-chain LBFs previously developed by Koehl and co-workers.^20^ The efficacy of these lipophilic salt LBFs was evaluated by analyzing the physicochemical properties and biorelevant *in vitro* performance of the synthesized lipophilic salt, enabling rational and judicious selection of appropriate counterions in lipophilic salt preparation.

## 2 Methods and Materials

### 2.1 Chemicals and Materials

The venetoclax free base was purchased from Kemprotec Ltd. (United Kingdom). Sodium docusate, sodium octadecyl sulfate, sodium dodecylsulfate, sodium decyl sulfate, sodium dodecyl sulfonate, sodium octyl sulfate, sodium octyl sulfonate, anhydrous sodium sulfate, Kolliphor RH40, Tween 85, Tris-maleate, sodium chloride, and calcium chloride were all purchased from Merck (Ireland). Silver nitrate was purchased from Fischer (Ireland). Peceol and Capmul were gifted from Gattefosse (France). Fasted state simulated intestinal fluid (FaSSIF) powder was purchased from Biorelevant (United Kingdom). All solvents were of analytical grade and were purchased from Merck (Ireland) and used as received.

### 2.2 Methods

#### 2.2.1 Lipophilic Salt Synthesis

All venetoclax lipophilic salts were prepared following a general procedure. The venetoclax free base (1 mmol) and counterions presented in Table 1 (1 mmol) were accurately dissolved in 160 mL of a biphasic solution of dichloromethane and water (1:1). 1 mmol methanolic HCl was added to the organic phase. The resulting mixture was stirred vigorously at ambient temperature overnight. This biphasic mixture was transferred to a separating funnel, and the organic phase was collected. The aqueous phase was further extracted by using dichloromethane. The combined organics were backwashed with cold distilled water until a negative silver nitrate (0.02 M aq) precipitate test result was obtained. The organic solution was dried with anhydrous sodium sulfate, filtered, and concentrated in vacuo. The resulting material was recrystallized with methanol and placed under high vacuum for 48 h.

**Table 1:**
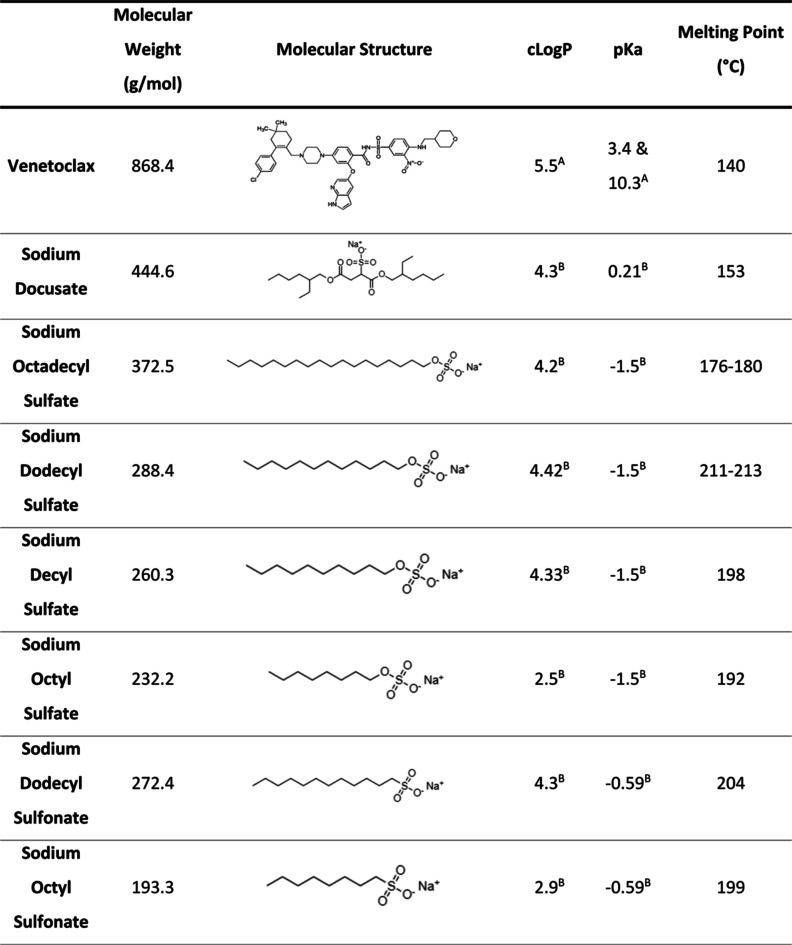
Table Containing a List of Physicochemical Properties Obtained of Venetoclax and Various Counterions Used in Lipophilic Salt Synthesis

a Koehl et al.^5^

b Calculated by ChemAxon.

#### 2.2.2 Thermal Analysis Using Differential Scanning Calorimetry

The melting peak temperature of venetoclax and venetoclax lipophilic salts were measured using a TA Q1000 with a TA Refrigerated Cooling system 90 (TA Instruments, Newcastle, DE). The cell was purged with nitrogen at a rate of 50 mL/min. 5 mg samples were weighed into T-zero pans (TA Instruments) and heated from 20 °C at a rate of 3 °C/min.

#### 2.2.3 Quantitative Analysis of Venetoclax with High-Performance Liquid Chromatography

Samples were analyzed using an Agilent 1200 series high-performance liquid chromatography (HPLC) system (Agilent Technology Inc., US) consisting of a binary pump, a degasser, an autosampler, and a variable wavelength detector. Data analysis was performed using EZChrom Elite version 3.2. A Zorbax Eclipse Plus C-18 column (5 μm, 4.6 mm × 150 mm × 70 Å) coupled with a Zorbax Eclipse Plus C-18 guard column (5 μm, 4.6 mm × 12.5 mm) was used to separate venetoclax from the sample. The mobile phase was composed of (a) acetonitrile with 0.5% (v/v) trifluoracetic acid and (b) water with 0.5% (v/v) trifluoroacetic acid at a ratio of 53:47 (v/v). A flow rate of 1 mL/min was used. The sample injection volume was 20 μL, and the detection wavelength was set to 316 nm. The column was set at a temperature of 37°. The purity of venetoclax lipophilic salts was calculated by obtaining a calibration curve of known concentrations of a venetoclax free base in its pure form. Known concentrations of the lipophilic salt form of venetoclax were then analyzed using HPLC, and the theoretical purity based on the equimolar ratio of the drug to counterion within the complex was used to calculate the free base equivalent within the salt complex.

#### 2.2.4 Nuclear Magnetic Resonance (^1^H and ^13^C NMR) Spectroscopy

(^1^H) NMR spectra of venetoclax and venetoclax lipophilic salts were recorded at 500 MHz, and ^13^C NMR spectra were recorded at 125.8 MHz on a Bruker Avance 500 NMR spectrometer using a Quattro NucleusProbe (QNP) at 300 K. All spectra were analyzed using Top-Spin 3.6.4 software. Spectra were obtained in deuterated dimethyl sulfoxide (DMSO-*d*_6_) and referenced to the residual solvent signal at 2.5 or 39.5 ppm for proton and carbon NMR spectroscopy, respectively. Chemical shifts (δH and δC) are expressed in parts per million (ppm), with positive shifts being downfield with regards to tetramethylsilane (TMS). Coupling constants (*J*) are expressed in hertz (Hz). Splitting patterns in ^1^H NMR spectra are denoted as s (singlet), d (doublet), t (triplet), q (quartet), quin (quintet), sext (sextet), sept (septet), and m (multiplet).

#### 2.2.5 Fourier Transform Infrared Analysis

Measurements were performed using a Fourier transform infrared (FTIR) spectrophotometer (Spectrum Two, PerkinElmer, UK) comprising a universal attenuated total reflection (UATR) unit. Spectra were determined between 400 and 4500 cm^–1^. The resolution was set at 1 cm^–1^. PerkinElmer Spectrum version 10.4 was used for data acquisition and analysis.

#### 2.2.6 Lipid-Based Formulation Preparation

A medium-chain LBF, a long-chain LBF, and a surfactant-only formulation that previously have been investigated *in vivo*(20) were utilized for the purpose of this study. The composition of these formulations can be found in Table 2 below.

**Table 2:** Composition of the LBFs Investigated^a^

formulation	excipients
medium-chain LBF	30% Capmul
	35% Tween 85
	35% Cremophor RH 40
long-chain LBF	30% Peceol
	35% Tween 85
	35% Cremophor RH 40
surfactant only formulation	50% Tween 85
	50% Cremophor RH 40

a These formulations are classified as a type III-A for the medium- and long-chain LBFs and a type IV formulation for the surfactant only formulation, as per the lipid formulation classification system (LFCS).^1,25^

The formulations were prepared by initially weighing individual excipients into glass vials. The excipients were mixed at 50 °C at 300 rpm for 30 min, and this was followed by overnight mixing at 200 rpm at 37 °C (Mixdrive 15 2MAG, Germany).

#### 2.2.7 Solubility Studies in Lipid Excipients

Venetoclax and venetoclax lipophilic salt solubilities were determined in the range of excipients used for LBFs, as detailed in Table 2. An excess amount of venetoclax or venetoclax lipophilic salt was added to 2 g of the excipient or LBF in a screw-top glass vial. Samples were stirred at 200 rpm (Mixdrive 15 2MAG, Germany) at 37 °C. Samples were withdrawn after 24, 48, and 72 h, followed by centrifugation at 21,000*g* (Mikro 200R Andreas Hettich GmbH & Co. KG, Germany) for 30 min. The supernatant was extracted and centrifuged again under identical conditions. The resulting supernatant was solubilized 1:10 v/v in a mixture of acetonitrile and ethyl acetate (1:4 v/v), followed by a further 1:10 v/v dilution in acetonitrile and ethyl acetate (4:1 v/v). Samples were analyzed by HPLC as per the method above. All samples were analyzed in triplicate.

#### 2.2.8 Biorelevant Solubility

The media utilized in this experiment were composed of 2 mM Tris-maleate, 150 mM NaCl, and 1.06 mM CaCl_2_. The pH of the media was adjusted to pH 6.5. The media were supplemented with FaSSIF powder a day prior to the experiment, resulting in a composition of the final biorelevant media of 3 mM taurocholate and 0.75 mM phospholipids. This final solution will be referred to as the “biorelevant media” throughout this paper. The biorelevant media was left at room temperature at least 2 h prior to usage. Excess amounts of the venetoclax free base and lipophilic salts of venetoclax were added to 2 mL of biorelevant medium. The suspensions were placed in an oven at 37 °C and were stirred at 200 rpm. Samples were taken at time points of 3, 6, and 24 h and centrifuged at 21,380*g* (Mikro 200 R, Hettich GmbH, Germany) for 30 min at 37 °C. The resulting supernatant was transferred to a new sample tube and centrifuged again under identical conditions. Samples were diluted 1:10 (v/v) with the mobile phase before analysis. The samples were analyzed using an Agilent 1200 series HPLC system (Agilent Technology Inc., United States) via HPLC, as described above.

#### 2.2.9 *In Vitro* Lipolysis

The LBFs were assayed using the *in vitro* lipolysis method^25^ to assess the potential for formulations containing dissolved lipophilic salt to maintain the drug in a solubilized state as the formulation is dispersed and digested under simulated gastrointestinal conditions. Formulations utilized in this experiment were loaded at 80% equilibrium solubility as per Section 2.2.6, at drug doses outlined in Table 3.

**Table 3:** Dose Utilized for Venetoclax and the Respective Lipophilic Salts in the LBF for the *In Vitro* Lipolysis Experiment

	dose (mg/g)—medium-chain LBF	dose (mg/g)—long-chain LBF
venetoclax free base	5.5	6.5
venetoclax suspension	50	50
venetoclax docusate	48	22
venetoclax dodecyl sulfate	45	21
venetoclax decyl sulfate	36	18
venetoclax dodecyl sulfonate	45	20

A control of the venetoclax free base at 80% equilibrium solubility and a suspension of 50 mg/g were also formulated. The *in vitro* lipolysis experiments were performed using a pH stat apparatus (Metrohm AG, Herisau, Switzerland) comprising an 836 Titando, an 804 Ti Stand, a pH electrode (Metrohm), and two 800 Dosino 20 mL dosing units. The system was operated by Tiamo 2.5 software (Metrohm). The biorelevant medium described in Section 2.2.8 was used for this experiment. The pancreatin extract (8× USP) was reconstituted immediately prior to use by adding 5 mL of the biorelevant medium to 1 g of pancreatin, vortexed thoroughly, and centrifuged at 4000*g* for 15 min at 4 °C (Centrifuge 5702 R, Eppendorf, Germany); 4 mL of the resulting supernatant was recovered and immediately stored on ice before further usage. The LBF was introduced to the biorelevant medium in a ratio of 1:40 (v/v) and stirred for 15 min to disperse the formulation. Medium pH was automatically adjusted to pH 6.5, and the digestion was initiated through introduction of the lipase. A total volume of 40 mL of digestion media was reached following the addition of pancreatic lipase. The pH was maintained at pH 6.5 throughout the experiment by automatic titration with 0.6 M NaOH for the medium-chain formulations and 0.2 M NaOH for the long-chain formulations. The amount of NaOH dispensed was recorded by the system and used to assess the rate and extent of digestion. After 60 min of digestion, the pH was back-titrated to pH 9 to determine the release of nonionized free fatty acids. Samples of 1 mL were withdrawn at 5 and 10 min during the dispersion phase. Pancreatin was then added, and samples of 1 mL were again withdrawn at 5, 10, 20, 40, and 60 min during the digestion phase. Each sample was immediately treated with 1 M 4-bromophenylboronic acid in methanol (5 μL/mL) to terminate the digestion process. The samples were kept at 37 °C until centrifugation. The samples were centrifuged at 37 °C and 21,000*g* for 30 min using a benchtop centrifuge (Mikro 200 R, Hettich, Germany) to separate the drug that precipitated out of the solution and that that remained solubilized. As the formulation is a type III-A formulation, ultracentrifugation was not required as the lipids should readily disperse within the biorelevant medium.^25^ 100 μL of the supernatant was withdrawn and diluted in the mobile phase in a ratio of 1:10. If any undissolved particles appeared, these samples were additionally centrifuged for 5 min at 6000*g* (Mikro 200R, Hettich, Germany) to remove undissolved particles, and the supernatant was collected for analysis as described below. In the event of a venetoclax-rich aqueous layer being present, this was removed and diluted as per the solubilization of lipids from Section 2.2.7. This was then subsequently diluted in the mobile phase at a ratio of 1:10, and HPLC analysis was performed as per Section 2.2.3.

#### 2.2.10 Saturation during *In Vitro* Testing

The degree of saturation of drug concentrations in the aqueous phase during the *in vitro* lipolysis experiment was measured by comparing the measured aqueous phase concentration to the equilibrium drug solubility in samples containing dispersed and digested nondrug loaded formulations under identical conditions. Excess drug or lipophilic salt was added to 2 mL samples of the blank aqueous phase and stirred at 37 °C for 24 h. Samples were withdrawn, centrifuged, and analyzed under conditions identical to those in Section 2.2.9. The saturation ratio was calculated by using eq 1
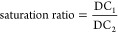
1where DC_1_ represents the aqueous concentration of the venetoclax free base or lipophilic salt during the *in vitro* dispersion and digestion test and DC_2_ represents the equilibrium concentration of the corresponding venetoclax free base or lipophilic salt measured in the blank aqueous phase (i.e., where the aqueous phase was obtained from the digestion of a blank LBF). Saturation ratios were evaluated at the end of the dispersion phase and the end of the digestion phase.

## 3 Results

### 3.1 Characterization of Venetoclax and Venetoclax Lipophilic Salts

The reaction of the venetoclax free base with the alkyl sulfate and sulfonate counterions in the presence of methanolic HCl yielded the corresponding salt form in all cases, forming bright yellow solids. FTIR spectroscopy was performed to confirm the presence of sulfate and sulfonate functional groups. FTIR spectroscopy (see Figures S1–S8, Supporting Information) confirmed characteristic bands at 1204 cm^–1^ (S=O stretch) and 2932 and 2859 cm^–1^ (C–H stretching) of the alkyl counterions and venetoclax free base, respectively. The spectra of venetoclax docusate contained a sharp band at 1735 cm^–1^, indicative of C=O stretch in the carboxylic acid group, and C=O ester of the docusate counterion. Similarly, spectra for venetoclax alkyl chain salts displayed increased intensity in the sulfate–sulfonate region of the FTIR spectra. ^13^C and ^1^H NMR (see Figures S9–S23, Supporting Information) confirmed the salt formation and ratio of venetoclax to the counterion. The signal at 14 ppm in the ^13^C spectrum indicates the presence of the sulfate/sulfonate functional group within the salt. Similarly, in the ^1^H spectrum, the most upfield signal at 0.85 ppm is observable and indicates the presence of the CH_3_ terminal present in the alkyl anion. The methine proton of the tetrahydropyran ring in venetoclax generated a peak at 1.9 ppm, and this peak was used to confirm the presence of venetoclax and the 1.1 ratio between the active pharmaceutical ingredient (API) and counterion. For the ratio identification of the alkyl sulfates in ^1^H NMR spectra, protons present at the CH_3_ terminal were used, which had a chemical shift of 0.85 ppm and were clearly distinguishable from other protons in the venetoclax lipophilic salt spectra.

Venetoclax lipophilic salts demonstrated purity in excess of 97% using HPLC in all cases (Table 4). The high levels of purity indicate that a salt with a 1:1 ratio was formed between venetoclax and its respective counterions. This result is in agreement with the results of the NMR, where integration of the peaks gave a 1:1 molar ratio between the free base form of the API and the acidic counterion when compared to the expected molecular formula.

**Table 4:** Percentage Purity of Venetoclax and Venetoclax Lipophilic Salts

	% purity venetoclax in salt complex (%)
venetoclax free base	100
venetoclax docusate	98.76
venetoclax octadecyl sulfate	99.14
venetoclax dodecylsulfate	98.91
venetoclax decyl sulfate	99.55
venetoclax octyl sulfate	97.31
venetoclax octanesulfonate	99.02
venetoclax dodecanesulfonate	97.02

The differential scanning calorimetry (DSC) results (see Table 5 and Figures S24–S30, Supporting Information) indicate that the salts formed displayed crystalline properties due to the presence of an endothermic melting peak with an absence of an exothermic recrystallization peak. Venetoclax octadecyl sulfate displayed the lowest melting point, likely due to the lengthy 18 carbon chain generating more intermolecular disruption than shorter alkyl chain counterions such as octyl sulfate and sulfonate. No significant difference in the melting point was apparent when counterions of identical chain lengths but with differing functional groups were compared, demonstrating that the disruption to intermolecular forces is chain length-mediated.

**Table 5:** Thermal Properties of Venetoclax and Venetoclax Lipophilic Salts

	melting point (range)
venetoclax free base	140 °C^a^
venetoclax docusate	151 °C (143–159 °C)
venetoclax octadecyl sulfate	88 °C (81–95 °C)
venetoclax dodecyl sulfate	91 °C (87–96 °C)
venetoclax decyl sulfate	101 °C (95–107 °C)
venetoclax octyl sulfate	109 °C (104–114 °C)
venetoclax dodecyl sulfonate	90 °C (85–95 °C)
venetoclax octyl sulfonate	112 °C (106–118 °C)

a Koehl et al.^5^

### 3.2 Equilibrium Solubility Studies in LBFs

**Figure 2:**
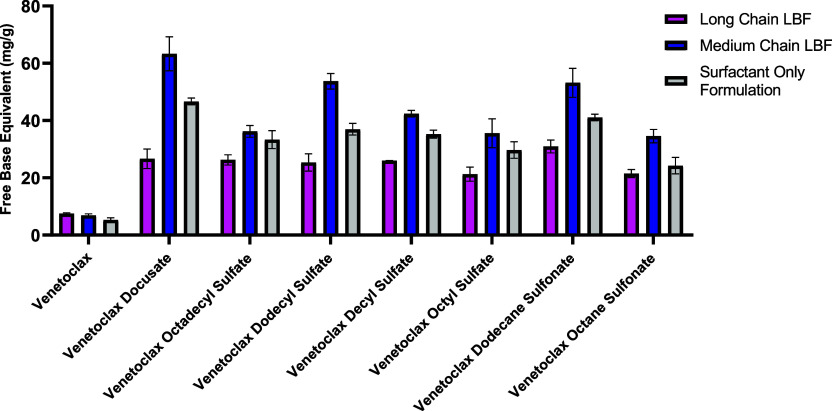
Equilibrium solubilities of the venetoclax free base and venetoclax lipophilic salt in both long- and medium-chain LBF. Data are expressed in venetoclax free base equivalents (mean ± SD, *n* = 3).

The solubility of the venetoclax free base and venetoclax lipophilic salts was assessed at 37 °C in both the medium- and long-chain LBF as well as a surfactant only formulation. All venetoclax lipophilic salts displayed a higher solubility in both medium- and long-chain LBF vehicles as well as in the surfactants only formulation when compared to the solubility of the free base form of venetoclax in the respective formulations (Figure 2). Venetoclax dodecyl sulfate, venetoclax dodecanesulfonate, and venetoclax docusate displayed the highest apparent solubility, >50 mg/g for medium-chain formulations and >25 mg/g for long-chain formulations. While with long-chain LBFs, all lipophilic salts displayed lower lipid solubility than their medium-chain counterpart, the gap in solubilities was the lowest for venetoclax octadecyl sulfate, demonstrating that the longer-chain alkyl chain within the salt complex led to a greater affinity for longer-chain excipients. The venetoclax free base displayed considerably lower solubility in both medium- and long-chain formulations with values of <7 mg/g reported for both. Venetoclax lipophilic salts also displayed up to a 6-fold increase in solubility in surfactant only formulations in comparison with the free base form of venetoclax. The high solubilities of the C_10_ and C_12_ alkyl chain salts in medium-chain formulations indicated the potential usage of both salts as an alternative to docusate; hence, the salts were progressed to the *in vitro* studies.

### 3.3 Solubility Testing in Biorelevant Media

All venetoclax lipophilic salts displayed higher solubility in aqueous media when compared to the aqueous solubility of the venetoclax free base (Table 6). The span of these solubility increases ranged from 0.35-fold in the case of venetoclax octanesulfonate to 1.9-fold for venetoclax docusate. No significant differences in solubility were observed in the cases of venetoclax alkyl sulfate and sulfonate lipophilic salts. This also demonstrates that in the absence of lipid excipients, lipophilic salts display only moderate solubility gains in biorelevant media.

**Table 6:** Biorelevant Solubility of Venetoclax and Venetoclax Lipophilic Salt at 37 °C (Mean ± SD, *n* = 3)

API/lipophilic salt	biorelevant solubility [μg/mL]
venetoclax	5.5 ± 1.0
venetoclax docusate	16.2 ± 1.6
venetoclax octadecyl sulfate	8.4 ± 0.7
venetoclax dodecane sulfate	10.9 ± 1.1
venetoclax decyl sulfate	10.3 ± 0.8
venetoclax octyl sulfate	7.7 ± 0.6
venetoclax octanesulfonate	7.4 ± 0.7
venetoclax dodecanesulfonate	9.8 ± 1.4

### 3.4 *In Vitro* Evaluation of Venetoclax Lipophilic Salt LBFs

**Figure 3:**
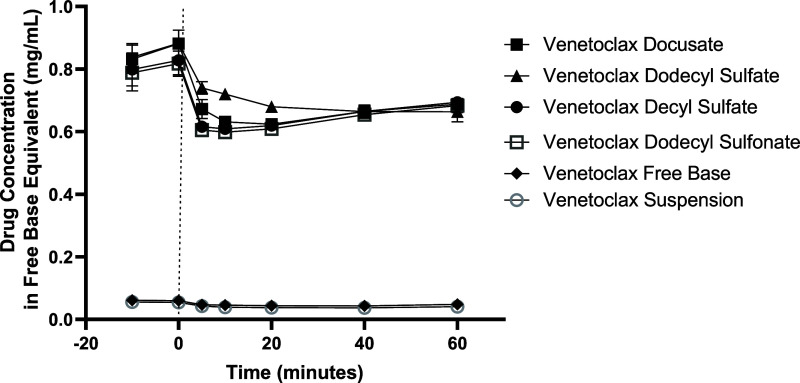
*In vitro* dispersion and digestion data of venetoclax lipophilic salt in type IIIA-MCF, where formulations were loaded at 80% of equilibrium solubility of the drug/salt complex, with the exception of the suspension that was loaded at 50 mg/g. The concentration of venetoclax lipophilic salt (in free base equivalents) in the aqueous phase of the medium-chain dispersion and digestion phases as a function of time. The dotted line indicates the end of the dispersion phase and the beginning of the digestion phase. Data are *n* = 3, mean ± SD.

**Figure 4:**
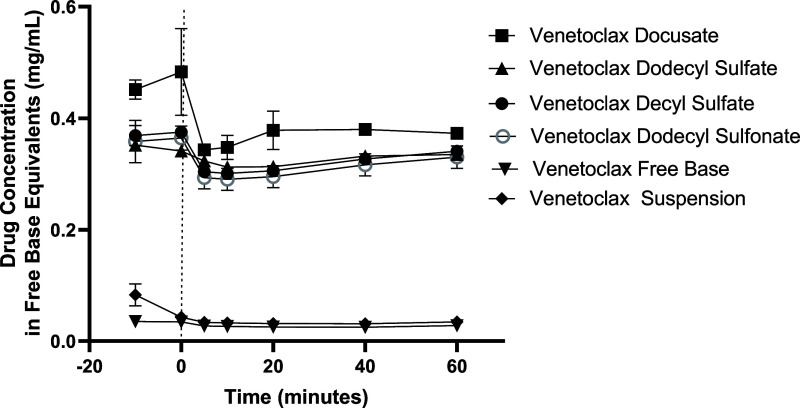
*In vitro* dispersion and digestion data of venetoclax lipophilic salt in Type IIIA-LCF, where formulations were loaded at 80% of equilibrium solubility of the drug/salt complex, with the exception of the suspension that was loaded at 50 mg/g. The concentration of venetoclax lipophilic salt (in free base equivalents) in the aqueous phase of the long-chain dispersion and digestion phases as a function of time. The dotted line indicates the end of the dispersion phase and the beginning of the digestion phase. Data are *n* = 3, mean ± SD.

**Figure 5:**
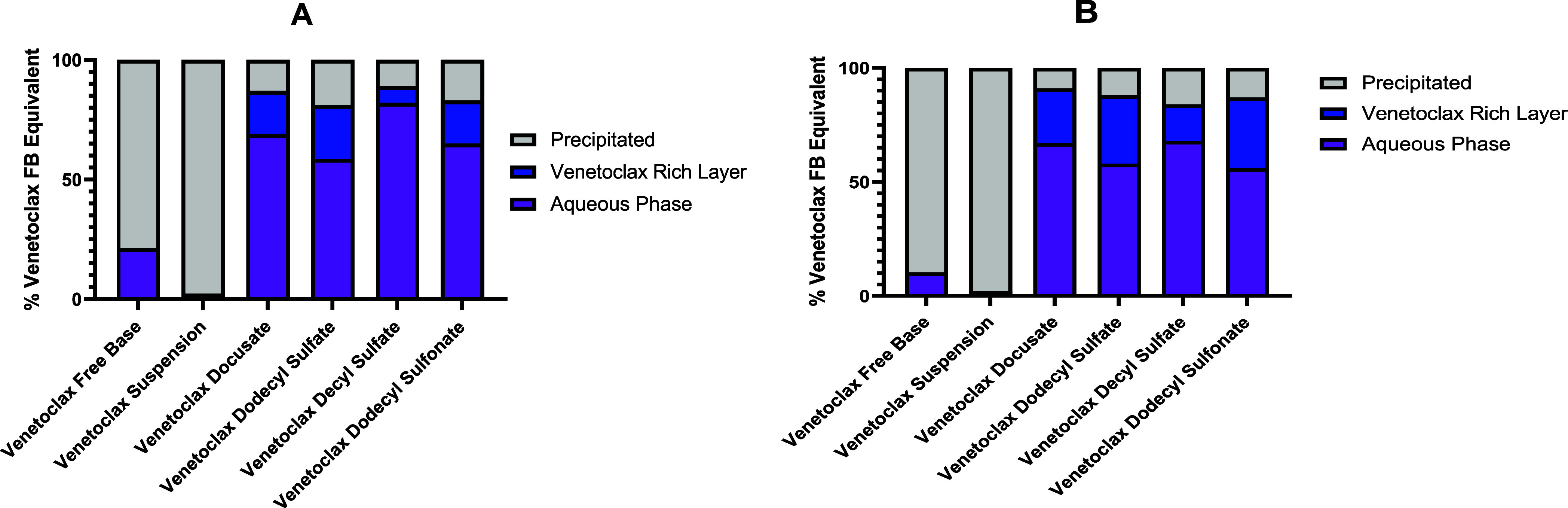
(A,B) Drug distribution in aqueous, pellet, and drug rich phases of venetoclax and the respective lipophilic salts of venetoclax in free base equivalents (FB) in medium- (A) and long-chain (B) formulations post digestion test. Dose % calculated as a factor of the total dose incorporated into the LBF as per Table 3.

The lipophilic salts formulated as LBFs were subsequently evaluated through *in vitro* lipolysis using a pH-stat apparatus following established procedures.^25^ The data demonstrate that an LBF containing venetoclax lipophilic salt was able to maintain venetoclax in a solubilized state upon dispersion and digestion of the formulation. Aqueous phase concentrations of up to ∼0.8 mg/mL were obtained for venetoclax lipophilic salts formulated as medium-chain LBFs after the dispersion phase, while aqueous phase concentrations for all venetoclax lipophilic salts achieved concentrations of ∼0.6 mg/mL after 60 min of digestion (Figure 3). For venetoclax lipophilic salts formulated as long-chain LBFs, concentrations of ∼0.5 and ∼0.4 mg/mL were obtained after the dispersion and digestion phase, respectively (Figure 4). For both medium- and long-chain LBFs, upon the initiation of digestion, a reduction in concentration in the aqueous phase was observed, and this most likely reflected digestion-induced precipitation of venetoclax from the colloidal dispersion. The extent of precipitation in long-chain LBFs was lower than that in medium-chain LBFs, as depicted in Figure 5A,B, which may reflect a lower extent of digestion of the longer-chain lipids^26^ Alternatively, it is possible that the higher solubilization capacity that was observed in solutions that contained digested long-chain lipid excipients relative to those that contained digested medium-chain counterparts also contributed to a lower extent of precipitation, and these observations have also been observed elsewhere.^27−29^ LBFs loaded with the venetoclax free base displayed considerably lower concentrations of venetoclax in the aqueous phase with concentrations of <0.05 mg/mL. The higher concentrations of venetoclax in the aqueous phase for all venetoclax salts than that in the free base demonstrate not only that the synthesis of the venetoclax lipophilic salt using a variety of counterions allows for much greater quantities of venetoclax to be dissolved in an LBF but that the lipophilic salt remains largely solubilized in the aqueous colloidal phase that forms during dispersion and digestion of the formulation.

Figure 5A,B demonstrates that high proportions of the dose of all venetoclax lipophilic salts were obtained from the aqueous phase after digestion (>60%). A venetoclax-rich layer was also observed in samples taken during the digestion phase of the lipolysis experiment and became apparent after centrifugation, and this had been reported previously for supersaturated LBFs of the venetoclax free base.^30^ While this layer was separate from the aqueous layer, it did not represent an increase in the solid phase; thus, up to 85% of the dose was in a molecularly dispersed state where the formulation contained a venetoclax lipophilic salt. As all lipophilic salt formulations were loaded at 80% equilibrium solubility, the molecularly dispersed solutions led to relatively similar proportions of precipitation between different salts. By comparison, greater than 80% of the dose present in venetoclax free base formulations precipitated after dispersion, with the venetoclax suspension displaying the highest proportion of precipitation, reflecting the highly unstable nature of the saturated formulation.

**Figure 6:**
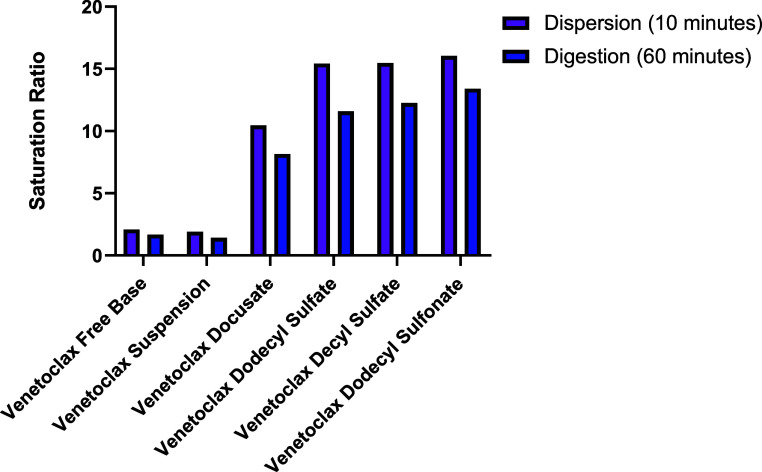
Saturation ratios for the medium-chain LBFs calculated from the aqueous phase concentrations (mg/mL) of the drug during *in vitro* dispersion and digestion tests after 10 min of the dispersion phase and 60 min of the digestion phase.

**Figure 7:**
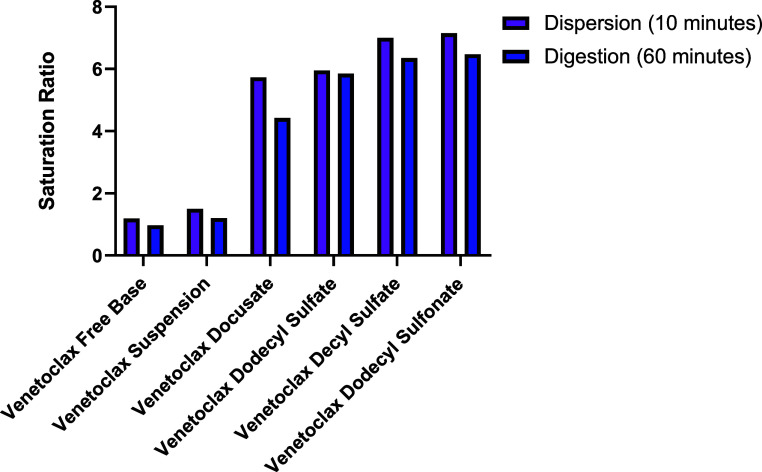
Saturation ratios for the long-chain LBFs calculated from the aqueous phase concentrations (mg/mL) of the drug during *in vitro* dispersion and digestion tests after 10 min of the dispersion phase and 60 min of the digestion phase.

Saturation ratios were calculated based on eq 1 and the results are presented in Figures 6 and 7. For salts loaded in medium-chain LBFs, saturation ratios ranged from ∼10 to ∼15 after dispersion and ∼8 to ∼14 after digestion, while salts loaded in long-chain LBFs had saturation ratios ranging from ∼6 to ∼7 after dispersion and ∼4 to ∼6 after digestion. This exemplifies that upon successful loading of a venetoclax lipophilic salt into lipid excipients with subsequent dispersion and digestion of the formulation, an increase in concentration of up 15-fold is observed in biorelevant media compared to that in solutions containing the blank LBF and nonformulated lipophilic salt. Venetoclax docusate displayed the lowest saturation ratio (<10.3 for all cases), which reflects its higher apparent solubility in biorelevant media, even in the absence of lipids (Table 6). A reduction in saturation ratios after the digestion phase compared to that after the dispersion phase was attributed to the increase in the precipitation of the sample, as well as the slightly higher solubility in the blank digested LBFs after digestion. The lower levels of saturation (<1.5) for both formulations containing the venetoclax free base illustrate the lower affinity of the venetoclax free base to the colloids present in solution than that of the lipophilic salts, resulting in a failure to retain the drug in a molecularly dispersed state and resulting in precipitation.

## 4 Discussion

A major limiting factor to the widespread application of LBFs is that many novel drug candidates display inherently poor lipid solubility.^3^ This realization has led to an increase in the development of strategies, such as lipophilic salt synthesis, to overcome the challenge of poor drug solubility in lipid excipients, allowing poorly soluble drugs to be formulated as LBFs. Previous work has demonstrated the utility of using docusate as a counterion in the formation of lipophilic salts of venetoclax as well as other poorly soluble drugs.^8,19,20,31,32^ However, docusate is a known stool softener and could potentially lead to dose-dependent laxative side effects.^22^ Thus, the purpose of this research project was to explore the versatility of other counterions in forming lipophilic salts of venetoclax and assess if comparable lipid solubility and *in vitro* performance to docusate could be obtained.

### 4.1 Physical Characteristics of Venetoclax Lipophilic Salts

Venetoclax lipophilic salts were successfully synthesized and isolated as yellow, crystalline lipophilic salts using a range of counterions. This study has demonstrated that using alkyl sulfates, sulfonates, and docusate, a range of suitable lipophilic salts can be synthesized using a robust method. Venetoclax salts synthesized in this study were solid at room temperature, demonstrating the difficulty presented in disrupting the venetoclax crystal lattice. DSC data indicated that all salts displayed degrees of crystallinity consistent with the lack of crystal lattice disruption, as indicated by sharp melting peaks. All salts utilizing alkyl chain counterions displayed a reduction in melting point, when compared to the free base form of venetoclax. In contrast, venetoclax docusate showed a slight increase in melting point, relative to its free base form. This result was attributed to the isolated polymorph upon crystallization of venetoclax docusate.^20^ It is not entirely clear as to why this did not affect the alkyl chain lipophilic salts, and an investigation into this was considered out of the scope of the current study. The physicochemical characteristics of the venetoclax molecule, such as its high molecular weight, planarity, and aromatic structure, are likely a key driver of this preserved crystalline structure. The development of lipophilic salts for a range of other drugs such as erlotinib, cabozantinib, itraconazole, halofantrine, cinnarizine lumefantrine, imidazolium ibuprofenate, and gefitinib has identified that a significant number of these developed compounds have been classified as ionic liquids rather than traditional lipophilic salts due to their melting points being below 100 °C. Such a low melting point indicates a considerable disruption of the salt’s crystalline structure, leading to higher solubilities. Many of these ionic liquids exhibit amorphous structures and remain liquid at room temperature, distinguishing them from typical crystalline lipophilic salt forms. However, some salts did display crystallinity, indicating that the solid-state form of the salt is both drug- and counterion-dependent.^8−10,12,32−34^

### 4.2 Enhanced Dose Loading of Lipophilic Salts in LBFs

Venetoclax lipophilic salts using alkyl chain and docusate counterions displayed enhanced solubilities in both medium- and long-chain LBFs when compared to those of the free base form of venetoclax. Overall, the solubility enhancements of the various lipophilic salts in lipid excipients were broadly comparable, highlighting the efficacy of alkyl chain counterions as suitable counterions in lipophilic salt synthesis. Of the lipophilic salts using the alkyl chain counterion, medium-chain (C_10_ and C_12_) counterions demonstrated the highest solubility in both medium- and long-chain formulations, attributed to their ability to disrupt the crystal lattice, with enhanced solubility in medium-chain lipids due to comparable alkyl chain lengths, promoting salt–excipient interactions. Additionally, hydrogen bonding between the drug and the ionic component of the lipophilic salt may have further enhanced solvation properties of venetoclax.^1^ While venetoclax octadecyl sulfate salts (C_18_), also displayed higher solubility in medium-chain LBFs, there was a smaller range in solubility between long- and medium-chain formulations when compared to other venetoclax salts. This higher solubility in long-chain formulations relative to that in the medium-chain formulations was attributed to the long 18-carbon chain counterion showing favorable interactions with long-chain LBFs (C_16_ and C_20_). Thus, increases in solubility for lipophilic salts in lipid excipients can be attributed to favorable interactions between similar chain length counterions and lipid excipients. In accordance with other studies, a trend was noticed whereby ionic liquids of gefitinib, cabozantinib, ceritinib, cinnarizine, and lumefantrine displayed higher solubilities in medium-chain LBFs than in long-chain LBFs.^8,34^

Melting point and solubility of the respective salt did not display a direct correlation. This was evident in the case of venetoclax octadecyl sulfate, which displayed a lower melting point and solubility than counterparts, likely influenced by intermolecular van der Waals forces between lipophilic salt molecules rather than interactions with excipients.^13^ Venetoclax docusate also displayed high lipid solubility despite its high melting point, indicating that the solubility of lipophilic salts in LBFs is not solely determined by the solid-state properties of the salt but also by their solvation characteristics and lipophilicity. DSC results showed that the salts retained crystallinity, and the improved solubility of venetoclax lipophilic salt in lipids is largely attributed to the increased lipophilicity rather than complete disruption of the crystal packing in the solid state. Therefore, the formation of venetoclax lipophilic salts appears to enhance the lipid excipient solubility primarily by strengthening solute–solvent interactions. Alkyl sulfates and sulfonates with the same chain length showed similar solubility in lipid excipients, demonstrating that sulfate verus sulfonate did not play a key role in enhanced lipid solubility but rather only in successfully forming the salt. Although formal stability testing has yet to be performed, preliminary observations did not reveal any macroscopic evidence of the precipitation or disproportionation of salts within the lipid excipients. This initial stability can likely be attributed to the adequately high Δp*K*_a_ difference between venetoclax and the counterions, which suggests the formation of a stable salt. However, long-term, formal stability studies would need to be conducted to assess the stability of the formulations upon storage and in the event of precipitation, and the addition of acidic excipients to the formulation could mitigate this risk.^35^ Despite this, the finding suggests that a variety of counterions could be effective, provided that the counterion used displays the suitable Δp*K*_a_ > 2 with the free form of the drug, allowing for formation of the salt.

### 4.3 *In Vitro* Assessment of Venetoclax Lipophilic Salt LBFs

The *in vitro* performance of venetoclax and venetoclax lipophilic salts formulated as LBFs was assessed using the dynamic *in vitro* lipolysis set up. The data suggest that both medium- and long-chain LBFs containing a range of venetoclax lipophilic salts were able to maintain venetoclax solubilization on dispersion and digestion in biorelevant media. Formulations containing the lipophilic salt form of venetoclax displayed superior *in vitro* performance compared to formulations loaded with the venetoclax free base. In the case of medium-chain LBFs, the aqueous concentrations obtained were broadly comparable, irrespective of the salt used. This observation was also noted that for long-chain LBFs, although concentrations here were lower than those for medium-chain LBFs, likely due to a lower initial dose loading. Up to 85% of the formulated dose remained in a molecularly dispersed state after dispersion and digestion for all salts and irrespective of the use of medium- or long-chain LBFs. This was significantly higher than formulations containing venetoclax free base where precipitation amounted to greater than 80% in all cases; thus, once incorporated into an LBF in a molecularly dispersed manner, it appears that despite digestion of the formulation, large portions of the drug can be retained in a solubilized manner due to the increased affinity of the salt for colloidal species and through integration into the aqueous phase in a predissolved state. The digestion of the formulation and subsequent release of free fatty acids may also serve to boost this concentration through the ionization of any dissociated venetoclax that displays basic properties,^36^ thus increasing its concentration in situ.

Significantly higher concentrations of venetoclax were obtained in biorelevant media containing dispersed and digested LBFs loaded with venetolcax lipophilic salts (Figures 3 and 4) than in the case of unformulated salts in nonlipid-containing biorelevant media (Table 6). This implies that while the formation of lipophilic salts increases solubility moderately in biorelevant media, the presence of lipid excipients can generate up to 80-fold increases in concentrations of the salt present in the biorelevant media. This increase in concentration can be attributed to the increased affinity of the lipophilic salt for colloidal species that are present during the dispersion and digestion of the LBF *in vitro* compared to that of the free base form of venetoclax. Incorporation of the lipophilic salt within an LBF followed by *in vitro* lipolysis led to large saturation ratio gains in biorelevant media relative to concentrations of drug in biorelevant media containing dispersed and digested blank LBFs (Figures 6 and 7). While these saturation ratios are quite high, they did not lead to substantial precipitation of venetoclax. Given that the free base form of venetoclax displays comparatively lower association with aqueous colloidal media, this suggests that complete salt dissociation of the lipophilic salt, leading to precipitation of the venetoclax free base in the aqueous media, is limited under these experimental conditions. This would therefore suggest that the benefit of lipophilic counterions not only improves dose loading in the LBF but also contributes to improved association of venetoclax within the aqueous colloidal media that form during digestion. This enhanced association could also be attributed to the fact that the counterions employed in this study can be used as anionic surfactants;^23^ thus, spontaneous micellar formation as well as enhanced incorporation of salts within mixed micelles may have occurred in solution during the dispersion and digestion tests, allowing for a greater solubilization capacity for venetoclax salt forms than for the free base form where the counterions were not present in solution.^10^ Overall, these results confirm a synergistic effect between incorporation of lipophilic salts in LBFs, leading to substantial concentration gains under biorelevant conditions relative to the venetoclax free base loaded as an LBF.

It must be noted that although saturation can be a driver of precipitation *in vitro*, increases in saturation *in vivo* can lead to increases in thermodynamic activity and thus may result in increases in absorption.^37,38^ As such, whether saturation results in an increase or a decrease in absorption is typically a trade-off between the drivers of precipitation and absorption. The *in vitro* data from Tay and colleagues suggest that the Type III formulations containing the lumefantrine ionic liquid, where both solubilization and saturation were maintained, like venetoclax, were most likely to promote absorption *in vivo*, particularly where an absorptive sink is present *in vivo*.^31^ In the case of cabozantinib and erlotinib ionic liquids, where significant precipitation was observed *in vitro*, it did not appear to significantly limit exposure of both drugs *in vivo*.^34^ Thus, the high levels of saturation recorded for venetoclax lipophilic salts were not a cause for concern, particularly given the low levels of precipitation observed during the dispersion and digestion of venetoclax lipophilic salt LBFs.

## 5 Conclusions

The data presented here have demonstrated that lipophilic salts in conjunction with an LBF can lead to significant solubility gains in lipid excipients and an improvement in the *in vitro* performance of venetoclax. This study has illustrated the versatility of alkyl sulfates and sulfonates as alternative counterions to docusate in lipophilic salt synthesis of venetoclax. Up to 9-fold higher solubility in medium- and long-chain prototype LBFs was obtained using venetoclax lipophilic salts when compared to the free base form of venetoclax. Thus, judicious selection of a counterion should be performed when selecting counterions for use in the synthesis of lipophilic salts. It must be realized that this selection is drug-dependent and studies would need to be conducted for each molecule as the physicochemical properties could be affected differently depending on the drug molecule in question. Overall, the results of this study highlight the potential to deliver venetoclax as an LBF in the lipophilic salt form using a range of counterions despite potentially poor inherent solubility.

## Supporting Information Available

Physicochemical characterization of all lipophilic salts of venetoclax; ^1^H and ^13^C NMR characterization data; and ^1^H and ^13^C NMR, FTIR, and DSC spectra (PDF)

## Abbreviations

API: active pharmaceutical ingredient
DSC: differential scanning calorimetry
FaSSIF: fasted state simulated intestinal fluid
FTIR: Fourier transform infrared
GRAS: generally recognized as safe
HPLC: high-performance liquid chromatography
LBF: lipid-based formulation
LFCS: Lipid Formulation Consortium System
NMR: nuclear magnetic resonance
UV: ultraviolet
