# Supplementary material for: Rationalizing Counterion Selection for the Development of Lipophilic Salts: A Case Study with Venetoclax
Source: Mol Pharm. 2024 May 4;21(6):2981–92. doi: 10.1021/acs.molpharmaceut.4c00106 (PMC11151202; doi:10.1021/acs.molpharmaceut.4c00106)
Supplement: Supplementary file 1 — mp4c00106_si_001.pdf [file mp4c00106_si_001.pdf]

Supporting Information

Rationalizing Counterion Selection for the Development of Lipophilic Salts: Case Study with Venetoclax

Physicochemical characterisation data of all lipophilic salts of venetoclax – including proton (^1H) and carbon-13 (^{13}C) NMR spectra, FTIR spectra and DSC thermograms for venetoclax free base and docusate, octadecyl sulfate, dodecyl sulfate, decyl sulfate, octyl sulfate, dodecyl sulfonate, and octyl sulfonate lipophilic salts of venetoclax.

Callum D. Ryan[a,b], Brendan T. Griffin[a,b], Joseph P. O'Shea[a*]

[a] School of Pharmacy, University College Cork, College Road, Cork, Ireland.

[b] SSPC, The Science Foundation Ireland Research Centre for Pharmaceuticals, School of Pharmacy, University College Cork, Ireland.

*Corresponding author
Email: joseph.oshea@ucc.ie
Tel: +353 -21-4901665

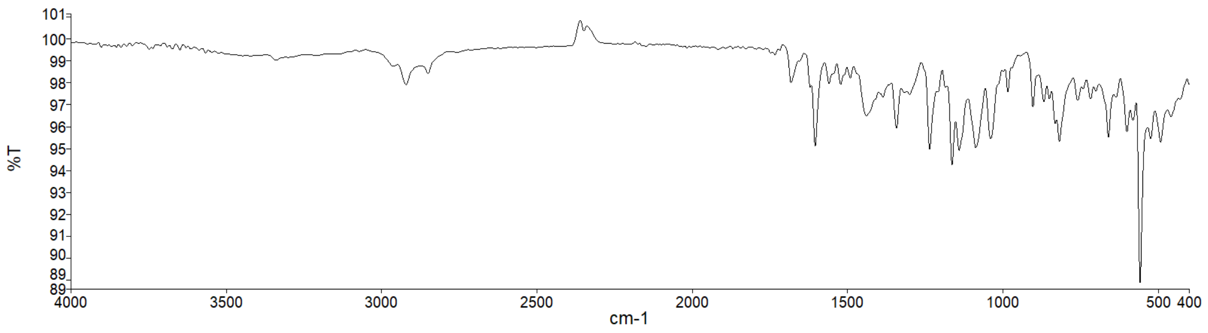

Figure S1: FT-IR plot for venetoclax free base.

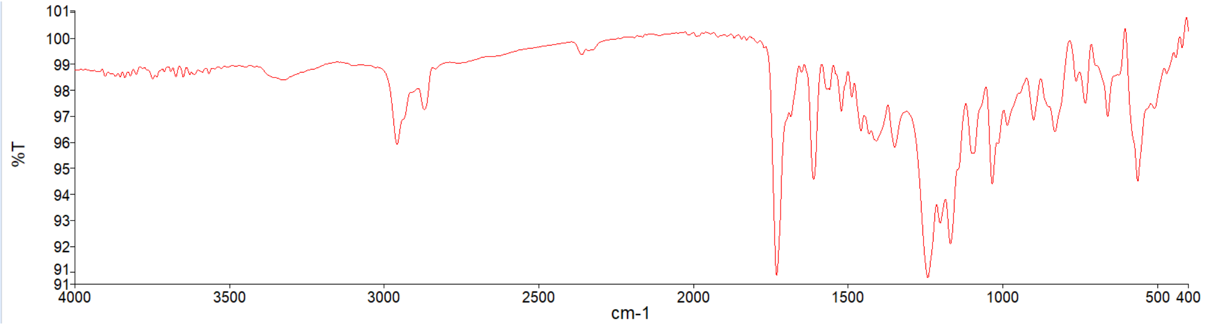

Figure S2: FT-IR plot for venetoclax docusate.

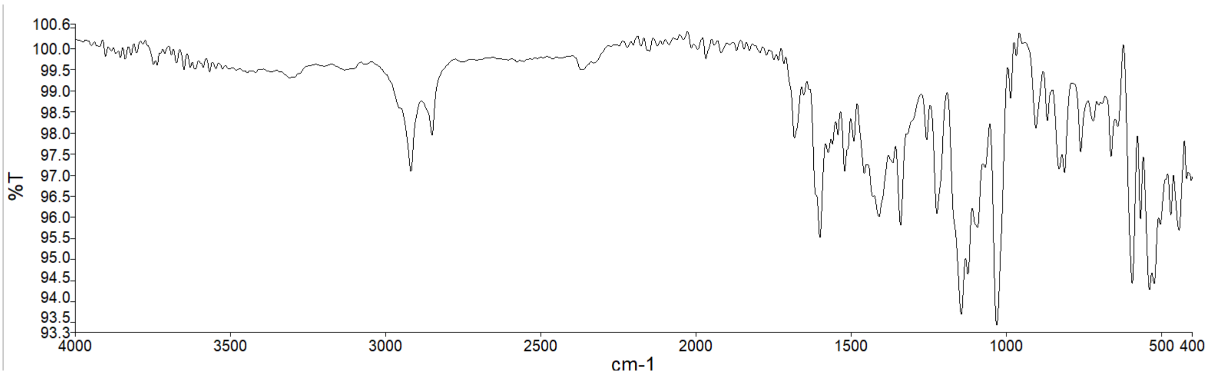

Figure S3: FT-IR plot for venetoclax octadecyl sulfate.

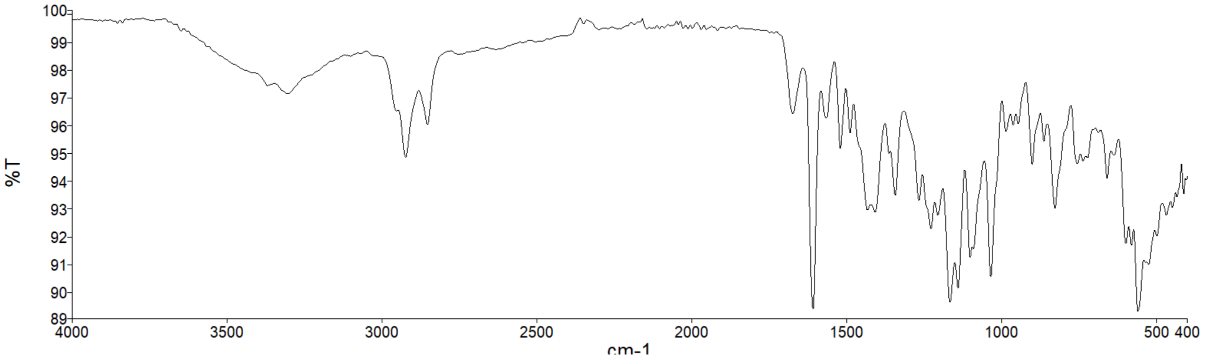

Figure S4: FT-IR plot for venetoclax dodecyl sulfate.

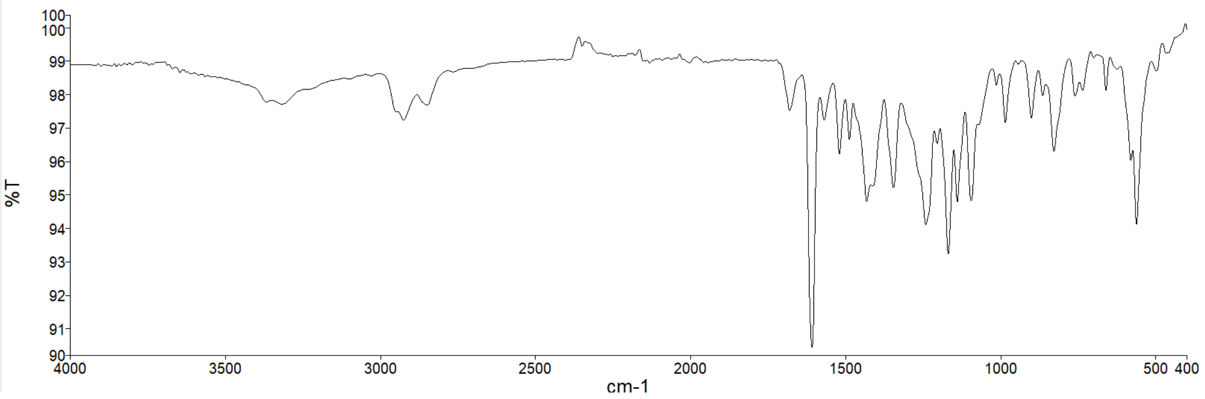

Figure S5: FT-IR plot for venetoclax decyl sulfate.

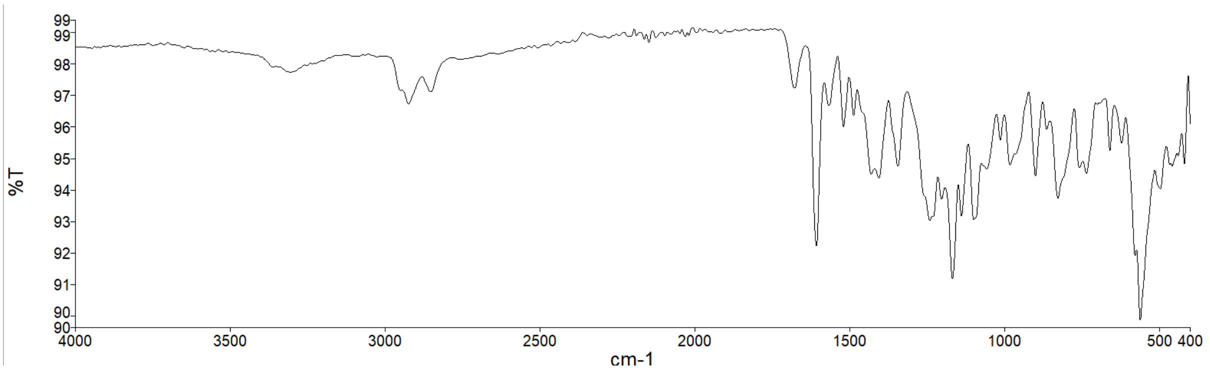

Figure S6: FT-IR plot for venetoclax octyl sulfate.

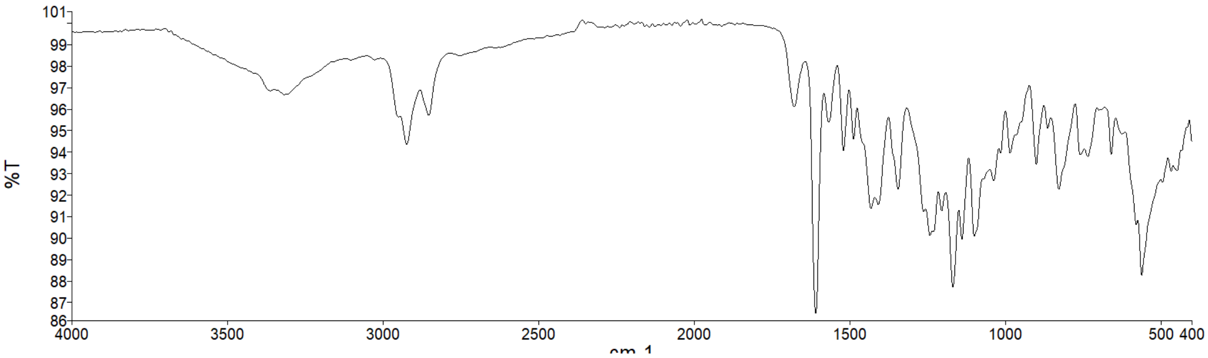

Figure S7: FT-IR plot for venetoclax dodecyl sulfonate.

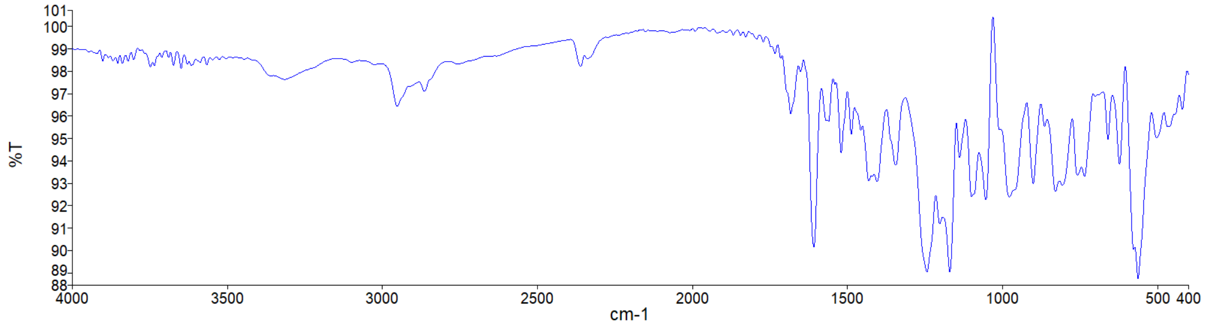

Figure S8: FT-IR plot for venetoclax octyl sulfonate.

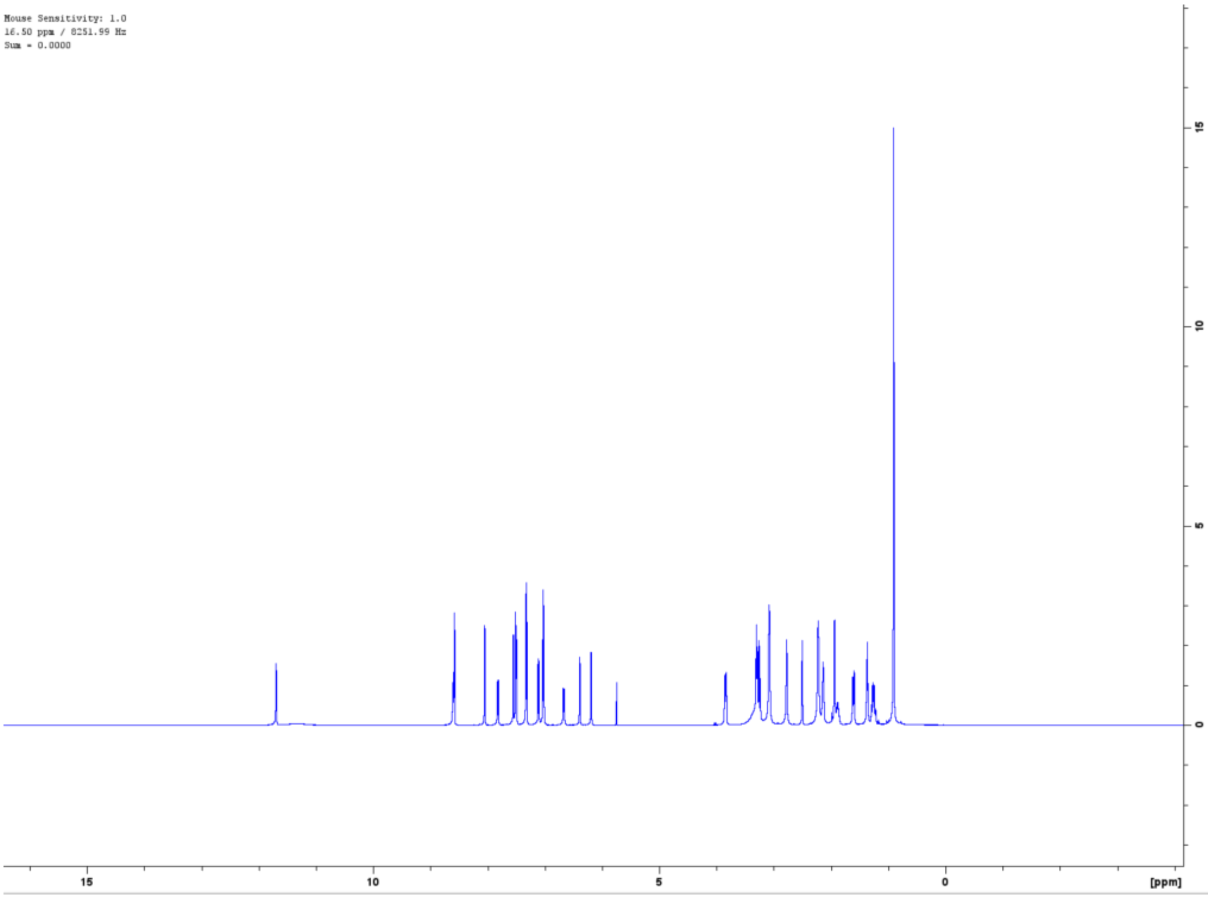

Figure S9: Venetoclax free base ^{1}H NMR in DMSO – d6 at 300K, 500 MHz δ 11.69 (t, J = 2.3 Hz, 1H), 8.55 – 8.64 (m, 2H), 8.05 (d, J = 2.6 Hz, 1H), 7.82 (dd, J = 2.3, 9.2 Hz, 1H), 7.55 (d, J = 2.6 Hz, 1H), 7.47 – 7.53 (m, 2H), 7.27 – 7.38 (m, 2H), 7.12 (d, J = 9.4 Hz, 1H), 6.99 – 7.06 (m, 2H), 6.67 (dd, J = 2.3, 9.1 Hz, 1H), 6.49 (dd, J = 1.9, 3.4 Hz, 1H), 6.19 (d, J = 2.3 Hz, 1H), 5.89 (s, 1H) 3.88 (ddd, j = 1.9, 4.5, 11.4 Hz, 2H), 3.21 – 3.34 (m, 5H), 3.08 (m, 4H), 2.87 (s, 2H), 2.22 (d, J = 6.1 Hz, 4H), 2.14 (d, J = 6.5 Hz, 2H), 1.94 (s, 2H), 1.88 (ddt, J = 3.9, 7.8, 11.3 Hz, 1H), 1.63 (ddd, J = 2.0, 4.1, 12.8 Hz, 2H), 1.36 (t, J = 6.5 Hz, 2H), 1.25 (qd, J = 4.5, 12.1 Hz, 2H), 0.92 (s, 6H).

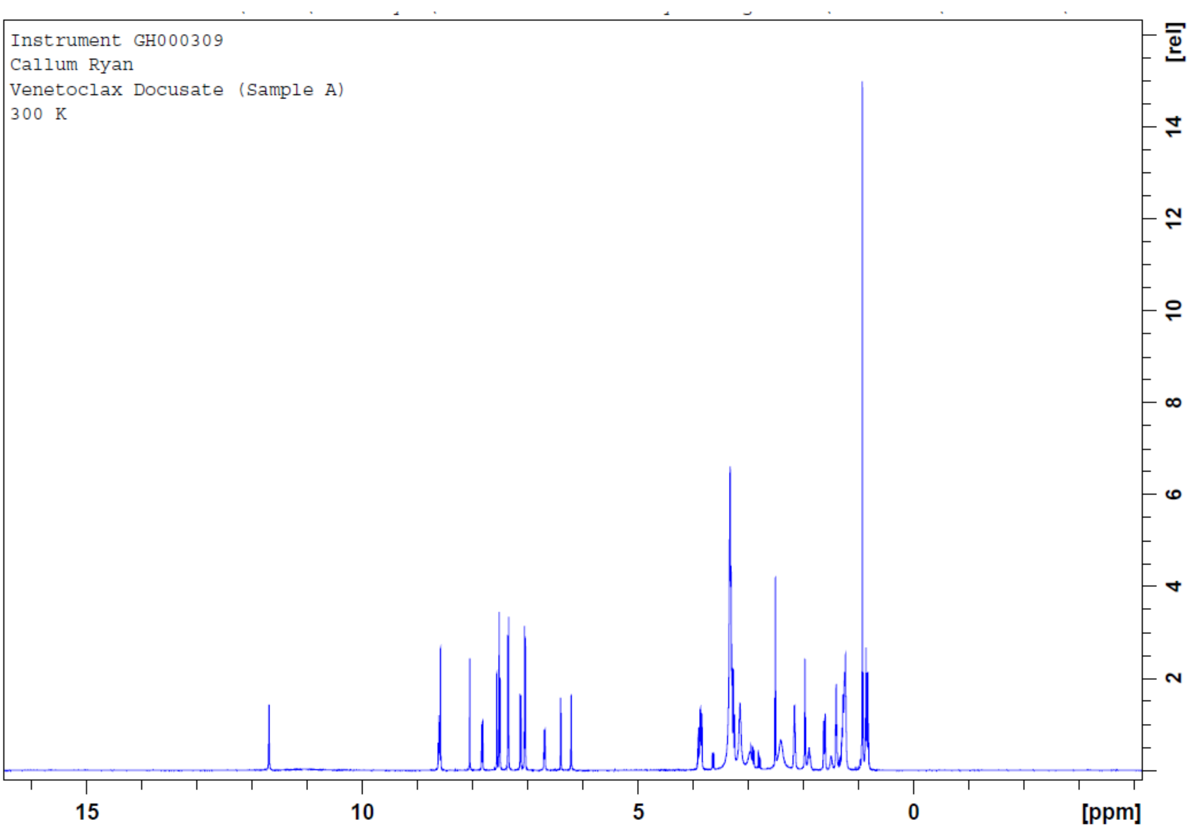

Figure S10: Venetoclax Docusate ^{1}H NMR in DMSO – d6 at 300K, 500MHz: δ 11.81 (s, 2H), 8.62 (m, 4H), 8.05 (s, 1H), 7.82 (dd, J = 1.7, 9.6 Hz, 1H), 7.56 (d, J = 2.5 Hz, 1H), 7.49 – 7.56 (m, 1H), 7.43 – 7.53 (m, 2H), 7.11 (dd, J = 8.9, 22.3 Hz, 2H), 6.72 (dd, J = 2.5, 8.4 Hz, 1H), 6.40 (dt, J = 3.7, 1.8 Hz, 1H), 6.26 (d, J = 2.1 Hz, 1H), 3.80 – 3.95 (m, 6H), 3.50 – 3.72 (m, 8H), 3.21 – 3.35 (m, 5H), 3.03 (t, J = 12.6 Hz, 2H), 2.92 (dd, J = 17.0, 11.8 Hz, 1H), 2.77 (m, 3H), 2.20 (t, J = 6.3 Hz, 2H), 2.02 (s, 2H), 1.89 (dqd, J = 3.3, 7.3, 11.1 Hz, 1H), 1.61 (d, J = 12.6 Hz, 2H), 65 1.47 (dt, J = 6.3, 12.8 Hz, 4H), 1.19 – 1.39 (m, 18H), 0.94 (s, 6H), 0.79 – 0.89 (m, 12H).

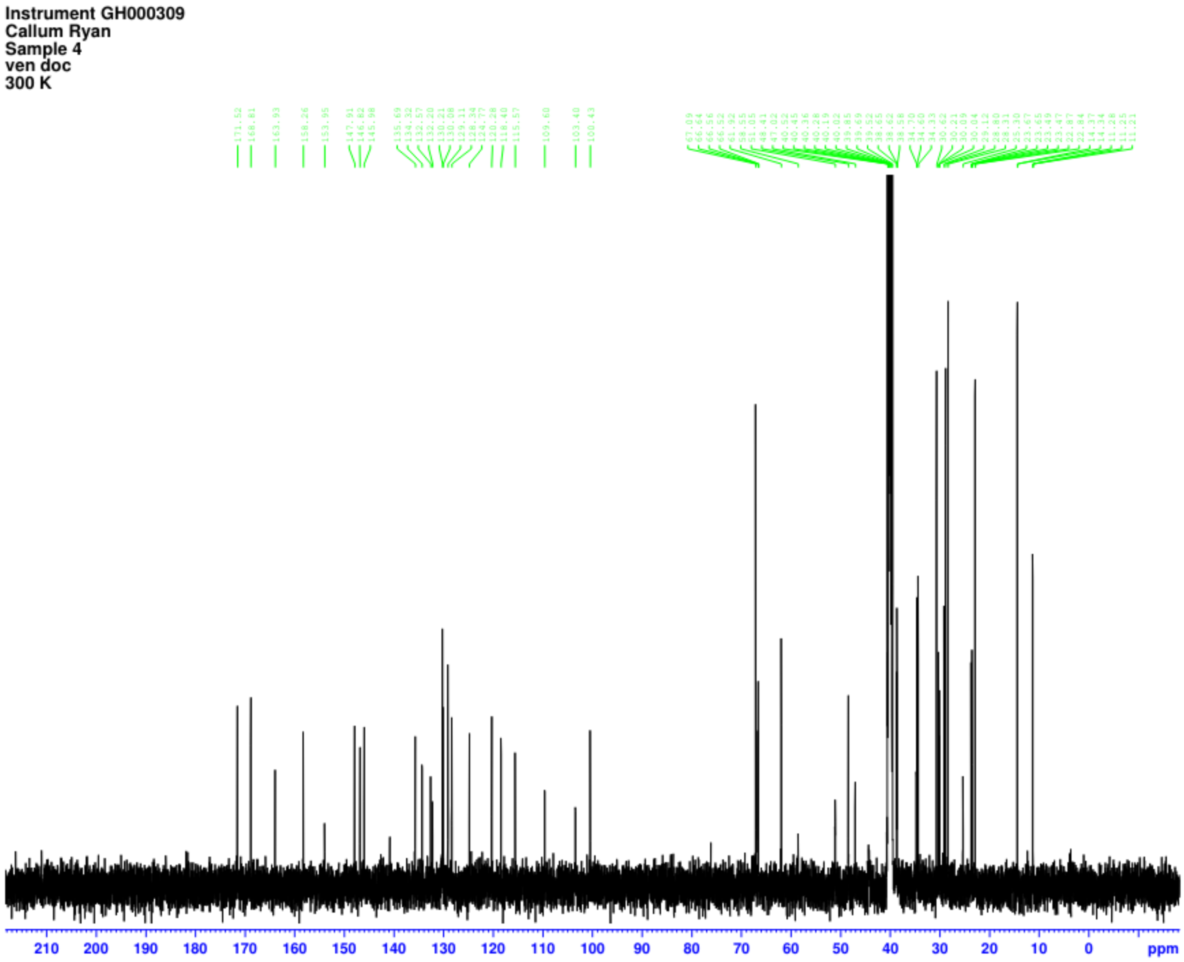

Figure S11: Venetoclax Docusate ^{13}C NMR in DMSO – d6 at 300K, 500 MHz: δ 171.52, 168.81, 163.93, 158.26, 153.95, 147.91, 146.82, 145.98, 135.69, 134.32, 132.57,132.20, 130.21, 130.08, 129.11, 128.34, 124.77, 120.28, 118.40, 115.57, 109.60, 103.40, 100.43, 67.09, 66.64, 66.56, 66.52, 61.92, 58.55, 51.05, 48.41, 47.02, 40.52, 40.45, 40.36, 40.28, 40.19, 40.02, 39.85, 38.65, 38.62, 38.58, 34.70, 34.60, 34.33, 30.62, 30.21, 30.09, 30.04, 29.12, 28.80, 28.31, 25.30, 23.67, 23.65, 23.49, 23.47, 22.87, 22.84, 14.37, 14.34, 11.12, 11.28, 11.21

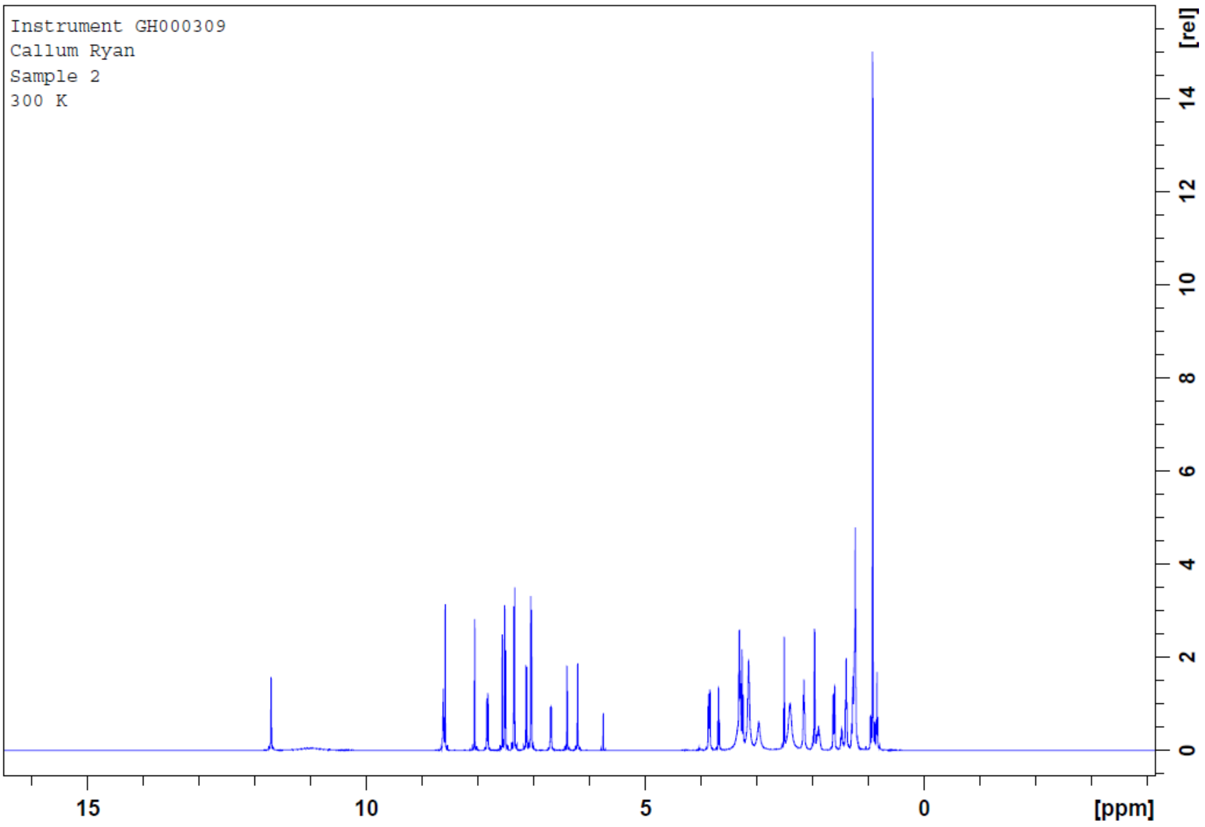

Figure S12: Venetoclax Octadecyl Sulfate ^{1}H NMR in DMSO – d6 at 300K, 500MHz: δ 11.66 (t, J = 2.1 Hz, 1H), 8.56 – 8.65 (m, 2H), 8.06 (d, J = 2.5 Hz, 1H), 7.84 (dd, J = 2.0, 8.8 Hz, 1H), 7.49 (d, J = 2.6 Hz, 1H), 7.47 – 7.53 (m, 2H), 7.27 – 7.39 (m, 2H), 7.12 (d, J = 9.4 Hz, 1H), 6.99 – 7.06 (m, 2H), 6.67 (dd, J = 2.3, 9.1 Hz, 1H), 6.35 (dd, J = 1.7, 3.3 Hz, 1H), 6.22 (d, J = 2.6 Hz, 1H), 5.81 (s, 1H), 3.89 (ddd, j = 2.0, 4.5, 11.4 Hz, 2H), 3.61 (s, 2H), 3.21 – 3.34 (m, 5H), 3.08 (t, J = 5.1 Hz, 4H), 2.77 (s, 2H), 2.22 (d, J = 6.1 Hz, 4H), 2.15 (d, J = 6.5 Hz, 2H), 1.93 (s, 2H), 1.88 (ddt, J = 3.9, 7.8, 11.3 Hz, 1H), 1.63 (ddd, J = 2.0, 4.1, 12.8 Hz, 2H), 1.36 (t, J = 6.5 Hz, 2H), 1.16 – 1.30 (m, 34H), 0.91 (s, 6H), 0.84 (m, 3H).

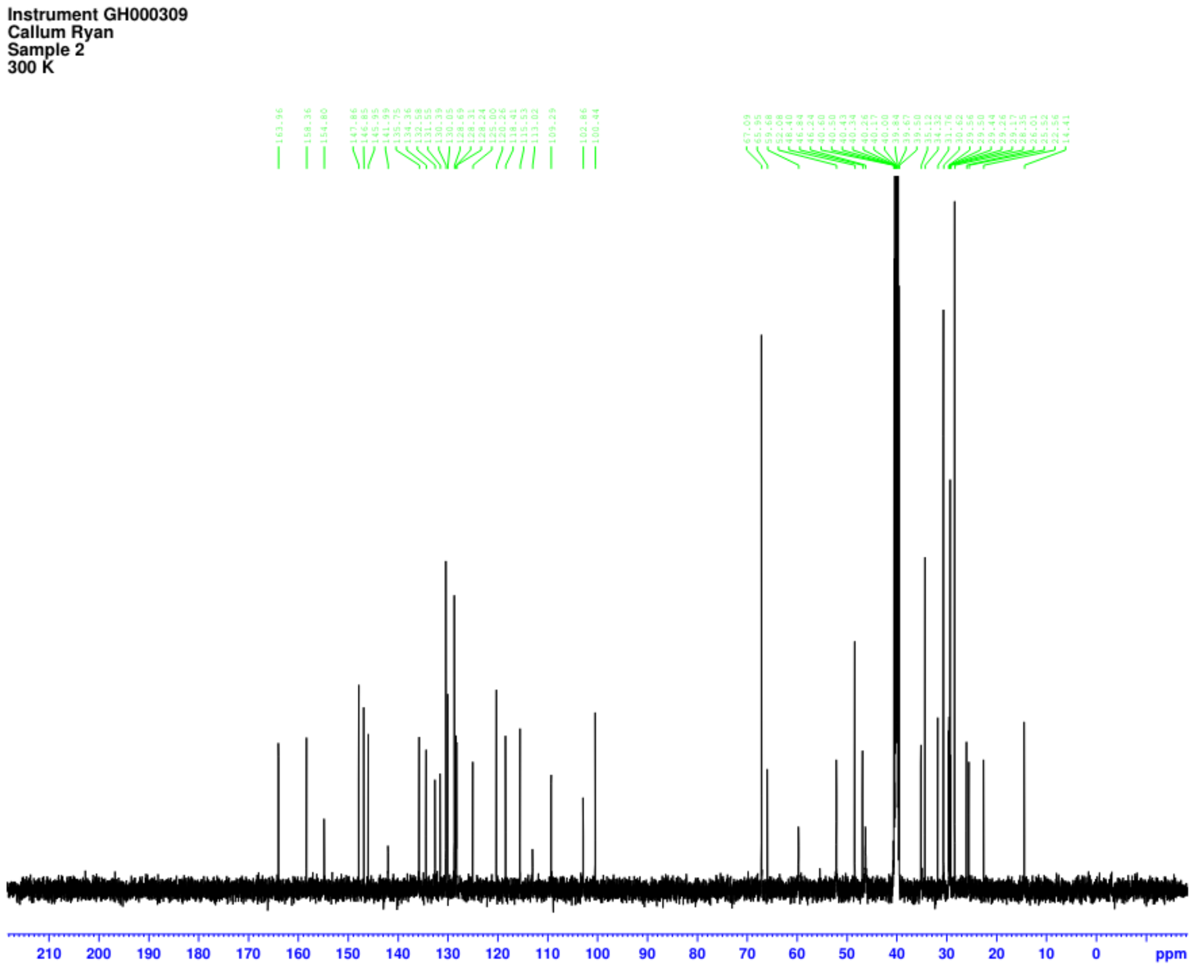

Figure S13: Venetoclax Octadecyl Sulfate ^{13}C NMR in DMSO – d6 at 300K, 500 MHz: δ 163.96, 158.36, 154.80, 147.86, 146.85, 145.95, 141.99, 135.75, 134.36, 132.58, 131.55, 130.59, 130.05, 128.69, 128.31, 128.24, 125.00, 120.26, 118.41, 115.53, 113.02, 109.29, 102.86, 100.44, 67.09, 65.95, 59.68, 52.08, 48.40, 46.84, 46.24, 40.60, 40.50, 40.43, 40.34, 40.26, 40.17, 40.00, 39.84, 39.67, 39.50, 35.13, 34.32, 31.76, 30.72, 29.57, 29.50, 29.44, 29.26, 29.17, 28.35, 26.01, 25.52, 22.56, 14.41

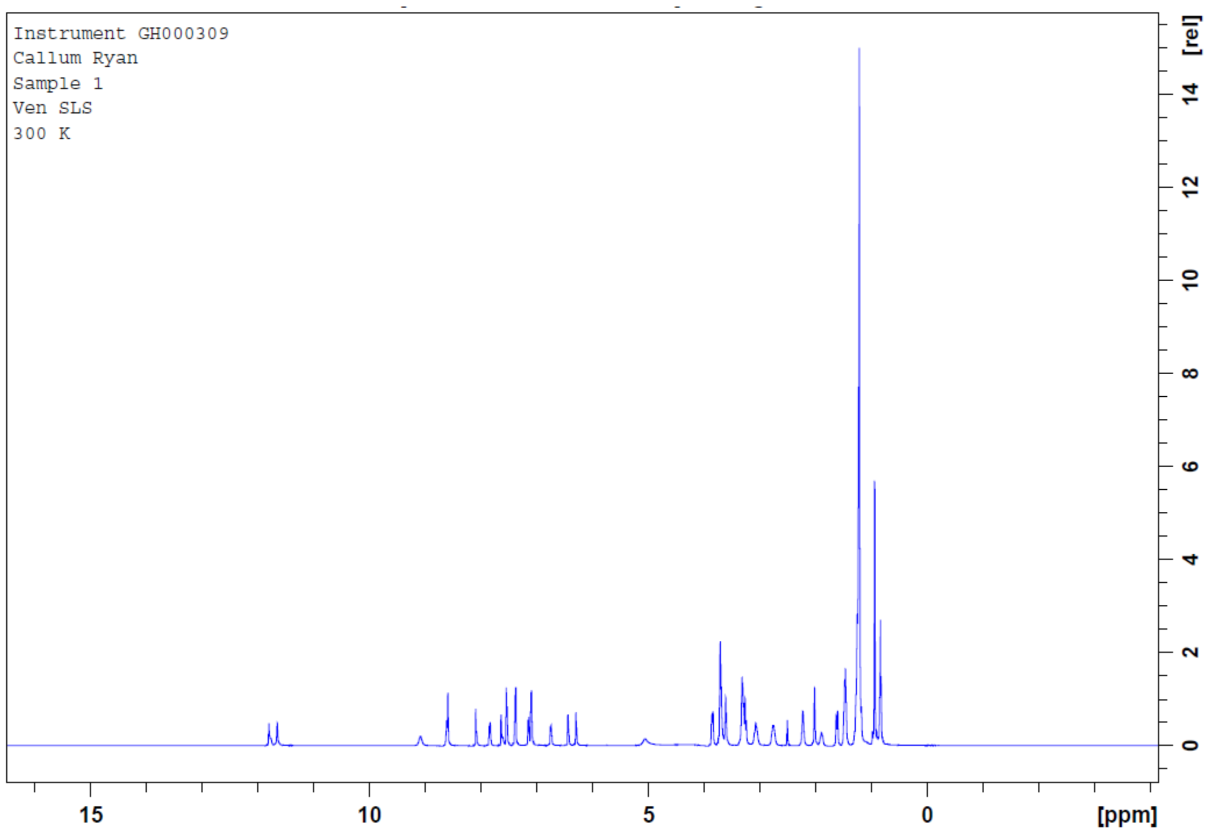

Figure S14: Venetoclax Dodecyl Sulfate ^{1}H NMR in DMSO-d6 at 300 K, 500 MHz: δ 11.82 (s, 1H), 11.64 (s, 1H), 8.56 – 8.65 (m, 2H), 8.06 (d, J = 2.5 Hz, 1H), 7.84 (dd, J = 2.0, 8.8 Hz, 1H), 7.49 (d, J = 2.6 Hz, 1H), 7.47 – 7.53 (m, 2H), 7.27 – 7.39 (m, 2H), 7.12 (d, J = 9.4 Hz, 1H), 6.99 – 7.06 (m, 2H), 6.67 (dd, J = 2.3, 9.1 Hz, 1H), 6.35 (dd, J = 1.7, 3.3 Hz, 1H), 6.22 (d, J = 2.6 Hz, 1H), 3.89 (ddd, j = 2.0, 4.5, 11.4 Hz, 2H), 3.61 (s, 2H), 3.25 – 3.33 (m, 5H), 3.08 (t, J = 5.1 Hz, 4H), 2.77 (s, 2H), 2.22 (d, J = 6.1 Hz, 4H), 2.15 (d, J = 6.5 Hz, 2H), 1.93 (s, 2H), 1.88 (ddt, J = 3.9, 7.8, 11.3 Hz, 1H), 1.63 (ddd, J = 2.0, 4.1, 12.8 Hz, 2H), 1.36 (t, J = 6.5 Hz, 2H), 1.17 – 1.30 (m, 22H), 0.91 (s, 6H), 0.84 (m, 3H).

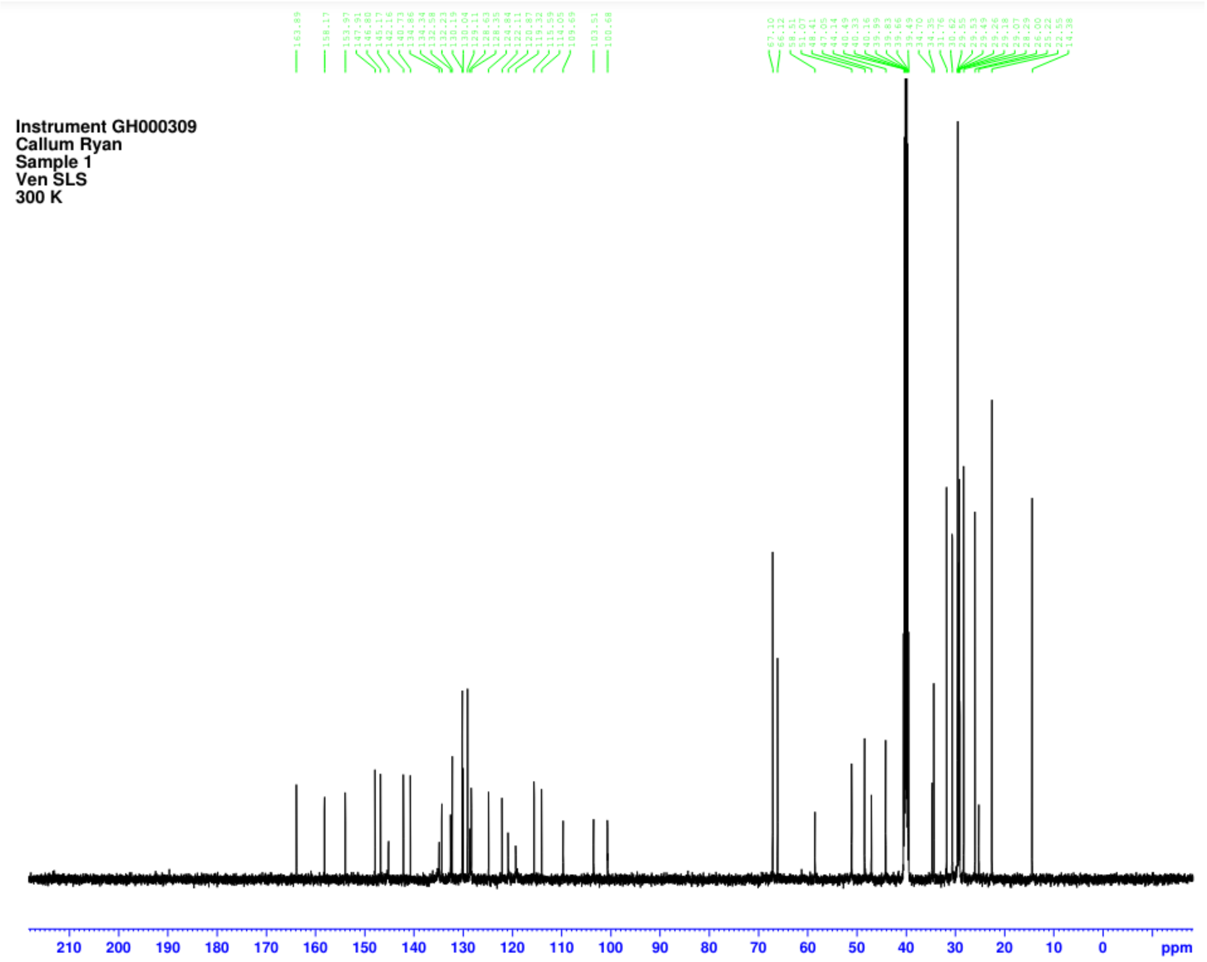

Figure S15: Venetoclax Dodecyl Sulfate ^{13}C NMR in DMSO – d6 at 300K, 500 MHz: δ 163.89, 158.17, 153.97, 147.91, 146.8, 145.17, 142.16, 140.73, 134.86, 134.34, 132.58, 132.23, 130.19, 129.11, 128.63, 128.35, 124.84, 122.11, 120.87, 119.32, 115.59, 114.05, 109.69, 103.51, 100.68, 67.10, 66.12, 58.51, 51.07, 48.41, 47.05, 44.14, 40.49, 40.33, 40.16, 39.99, 39.83, 39.66, 39.49, 34.70, 34.35, 31.76, 30.62, 29.55, 29.53, 29.49, 29.26, 29.18, 29.07, 28.29, 26.00, 25.22, 22.55, 14.38.

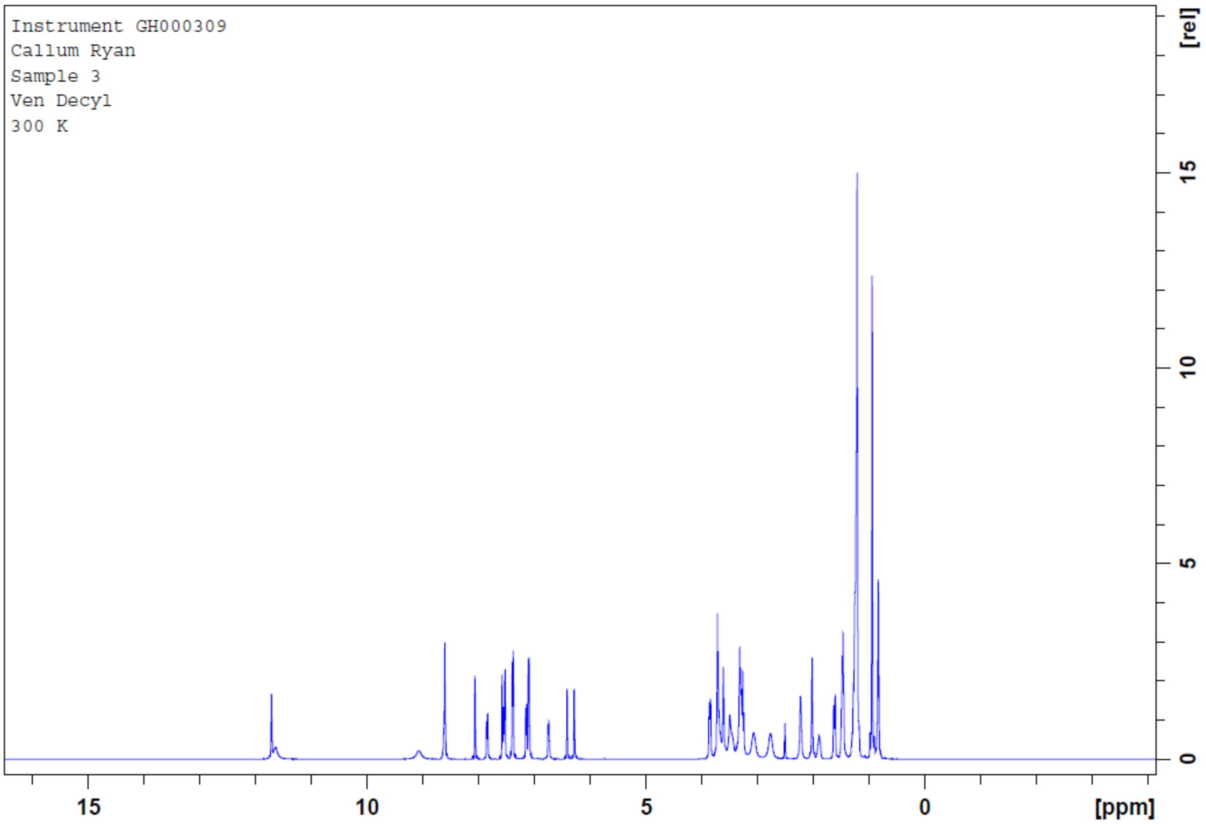

Figure S16: Venetoclax Decyl Sulfate ^{1}H NMR in DMSO-d6 at 300 K, 500 MHz: δ 11.72 (m, 1H), 8.56 – 8.65 (m, 2H), 8.06 (d, J = 2.5 Hz, 1H), 7.84 (dd, J = 2.0, 8.8 Hz, 1H), 7.49 (d, J = 2.6 Hz, 1H), 7.47 – 7.53 (m, 2H), 7.27 – 7.36 (m, 2H), 7.12 (d, J = 9.4 Hz, 1H), 6.98 – 7.05 (m, 2H), 6.61 (dd, J = 2.3, 9.2 Hz, 1H), 6.39 (dd, J = 1.9, 3.4 Hz, 1H), 6.22 (d, J = 2.6 Hz, 1H), 3.89 (ddd, j = 2.0, 4.5, 11.4 Hz, 2H), 3.61 (s, 2H), 3.25 – 3.33 (m, 5H), 3.08 (t, J = 5.1 Hz, 4H), 2.77 (s, 2H), 2.22 (d, J = 6.1 Hz, 4H), 2.15 (d, J = 6.5 Hz, 2H), 1.93 (s, 2H), 1.88 (ddt, J = 3.9, 7.8, 11.3 Hz, 1H), 1.63 (ddd, J = 2.0, 4.1, 12.8 Hz, 2H), 1.36 (t, J = 6.5 Hz, 2H), 1.12 – 1.29 (m, 18H), 0.94 (s, 6H), 0.84 (m, 3H).

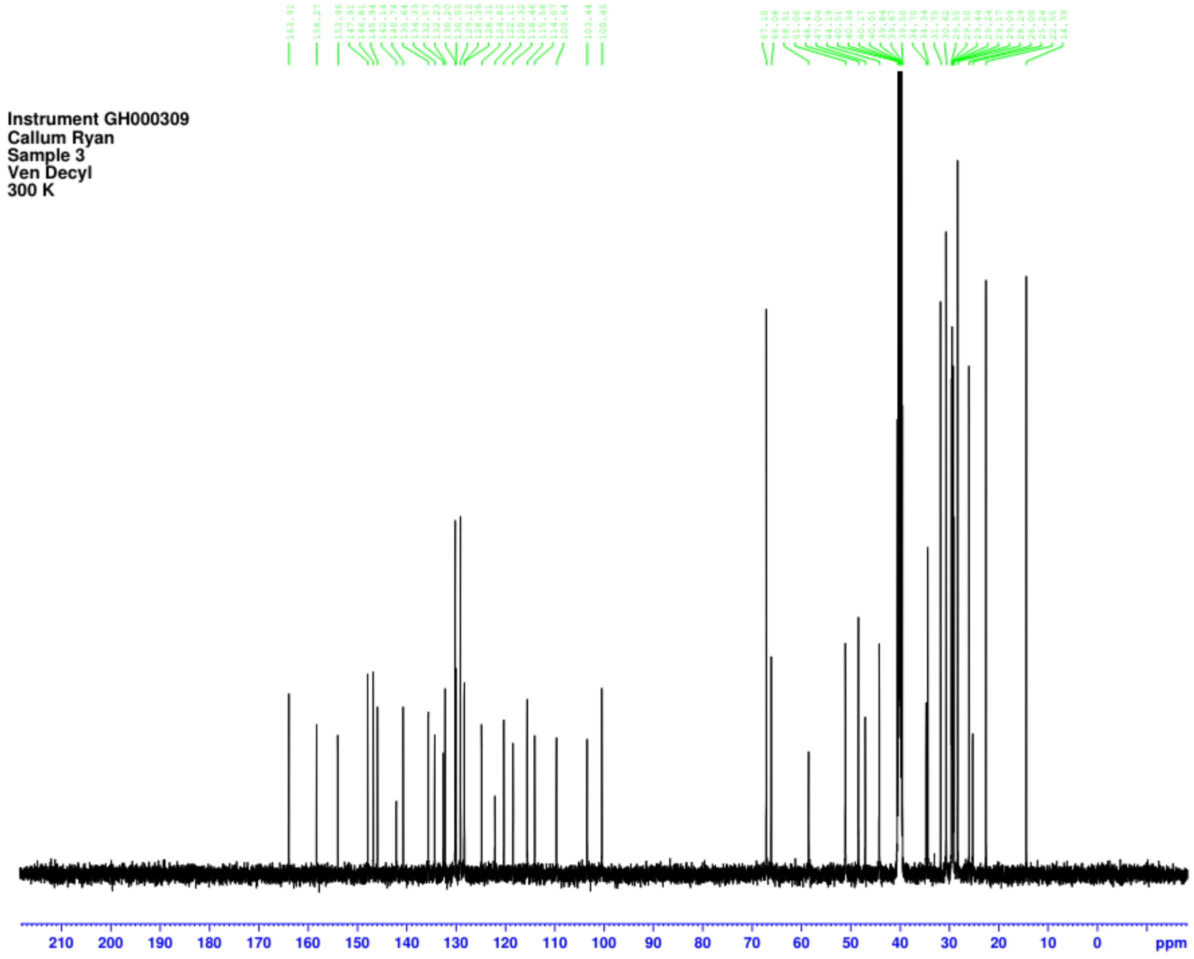

Figure S17: Venetoclax Decyl Sulfate ^{13}C NMR in DMSO – d6 at 300K, 500 MHz: δ 163.91, 158.27, 153.96, 147.91, 146.81, 145.94, 142.14, 140.74, 135.64, 134.33, 132.57, 132.23, 130.20, 130.05, 129.12, 128.36, 128.31, 142.82, 122.11, 120.32, 118.46, 115.58, 114.07, 109.64, 103.44, 100.45, 67.10, 66.08, 58.51, 51.06, 48.41, 47.04, 44.19, 40.51, 40.34, 40.17, 40.01, 39.84, 39.67, 39.50, 34.70, 34.34, 31.75, 30.62, 29.55, 29.50, 29.44, 29.24, 29.17, 29.09, 28.29, 26.00, 25.24, 22.55, 14.39

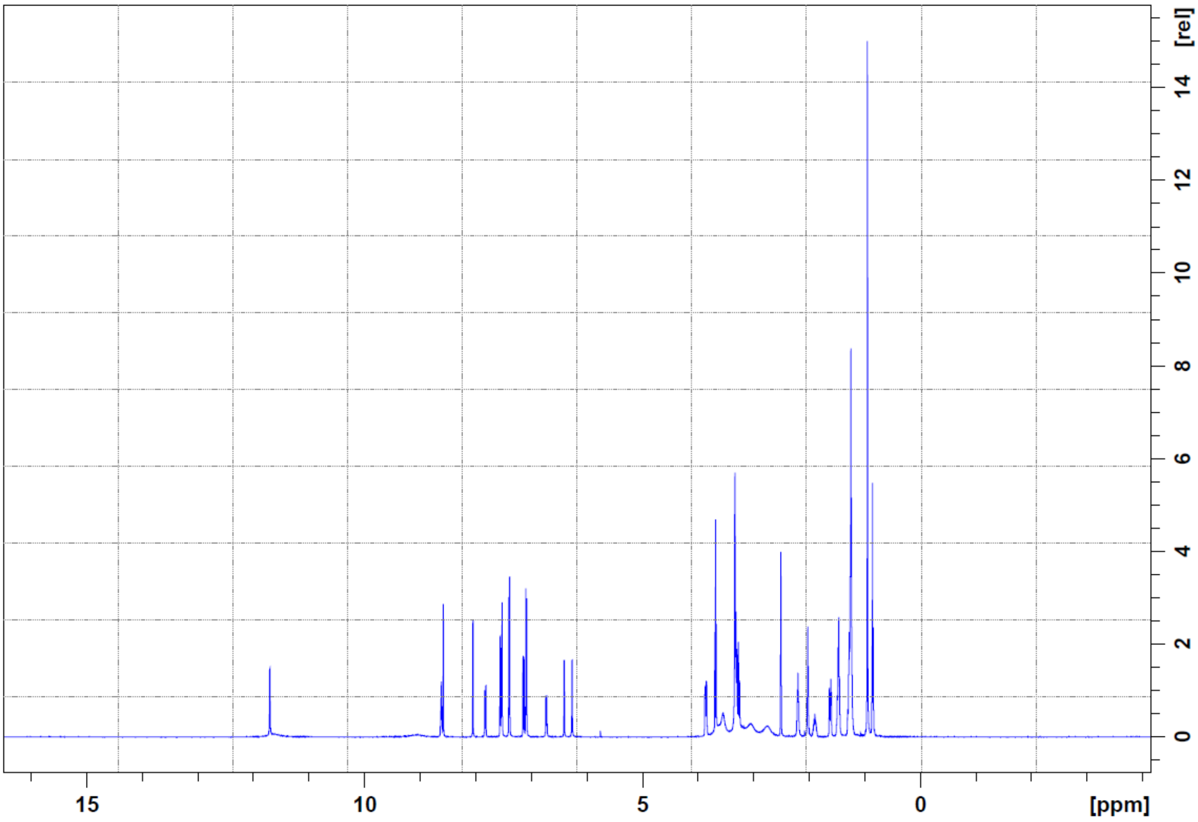

Figure S18: Venetoclax Octyl Sulfate ^{1}H NMR in DMSO – d6 at 300K, 500 MHz δ 11.70 (m, 1H), 8.56 – 8.65 (m, 2H), 8.06 (d, J = 2.5 Hz, 1H), 7.84 (dd, J = 2.0, 8.8 Hz, 1H), 7.46 (d, J = 2.8 Hz, 1H), 7.47 – 7.52 (m, 2H), 7.27 – 7.36 (m, 2H), 7.12 (d, J = 9.4 Hz, 1H), 6.98 – 7.05 (m, 2H), 6.61 (dd, J = 2.3, 9.2 Hz, 1H), 6.39 (dd, J = 1.9, 3.4 Hz, 1H), 6.22 (d, J = 2.6 Hz, 1H), 3.89 (ddd, j = 2.0, 4.5, 11.4 Hz, 2H), 3.61 (s, 2H), 3.25 – 3.33 (m, 5H), 3.08 (t, J = 5.1 Hz, 4H), 2.77 (s, 2H), 2.22 (d, J = 6.1 Hz, 4H), 2.15 (d, J = 6.5 Hz, 2H), 1.93 (s, 2H), 1.88 (ddt, J = 3.9, 7.8, 11.3 Hz, 1H), 1.63 (ddd, J = 2.0, 4.1, 12.8 Hz, 2H), 1.36 (t, J = 6.5 Hz, 2H), 1.15 – 1.33 (m, 14H), 0.94 (s, 6H), 0.84 (m, 3H).

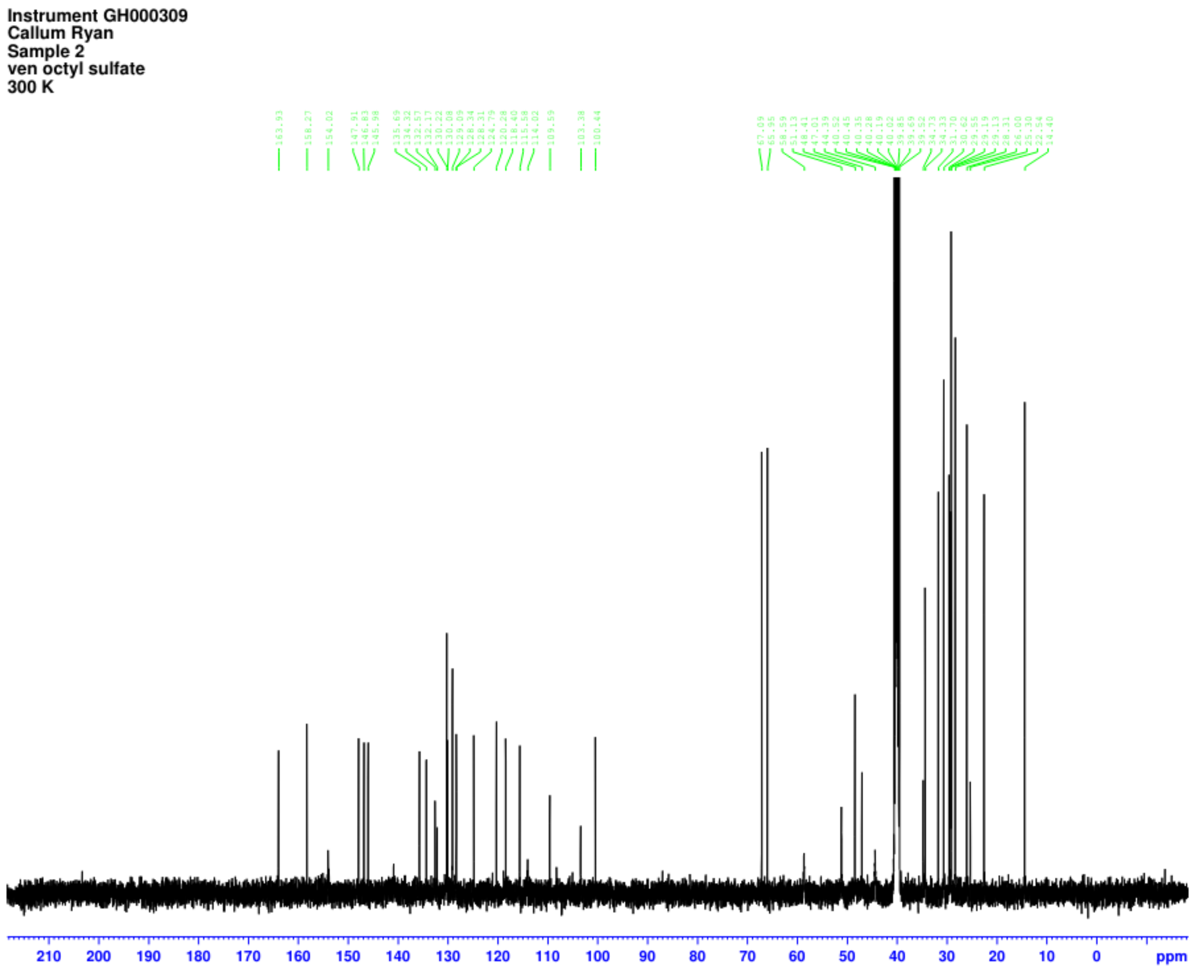

Figure S19 : Venetoclax Octyl Sulfate ^{13}C NMR in DMSO – d6 at 300K, 500 MHz: δ 163.93, 158.27, 154.02, 147.91, 146.83, 145.98, 135.69, 134.32, 132.57, 132.17, 130.22, 130.08, 129.09, 128.34, 128.31, 124.79, 120.28, 118.40, 115.58, 114.02, 109.59, 103.38, 100.44, 67.09, 65.95, 58.59, 51.13, 48.41, 47.01, 44.39, 40.52, 40.45, 40.35, 40.28, 40.19, 40.02, 39.95, 39.69, 39.52, 34.73, 34.33, 31.70, 30.62, 29.55, 29.19, 29.13, 28.31, 26.00, 25.30, 22.54, 14.40.

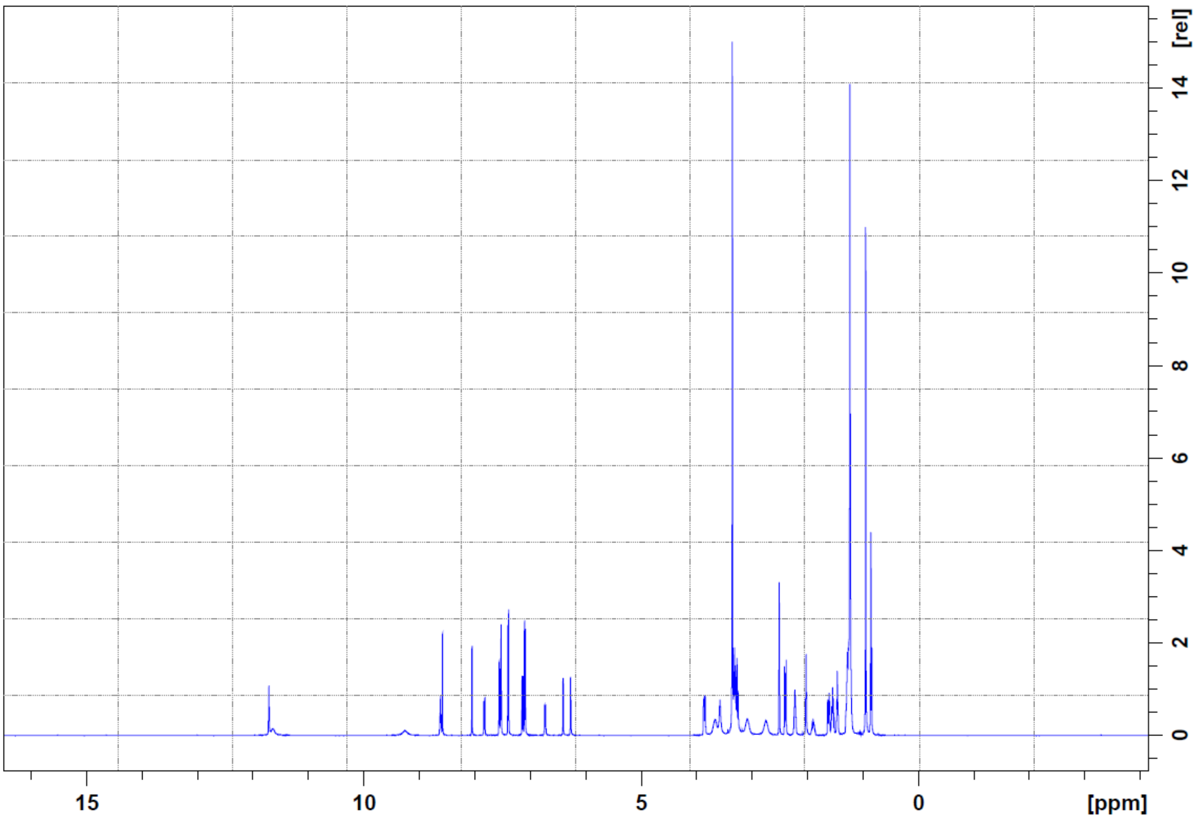

Figure S20: Venetoclax Dodecyl Sulfonate ^1H NMR in DMSO – d6 at 300K, 500 MHz δ 11.72 (m, 1H), 8.56 – 8.65 (m, 2H), 8.06 (d, J = 2.5 Hz, 1H), 7.84 (dd, J = 2.0, 8.8 Hz, 1H), 7.49 (d, J = 2.6 Hz, 1H), 7.47 – 7.53 (m, 2H), 7.27 – 7.36 (m, 2H), 7.12 (d, J = 9.4 Hz, 1H), 6.98 – 7.05 (m, 2H), 6.61 (dd, J = 2.3, 9.2 Hz, 1H), 6.39 (dd, J = 1.9, 3.4 Hz, 1H), 6.24 (d, J = 2.6 Hz, 1H), 3.99 (s, 1H), 3.89 (m, 2H), 3.03 – 3.63 (m, 14H), 2.77 (s, 2H), 2.22 (d, J = 6.1 Hz, 4H), 2.15 (d, J = 6.5 Hz, 2H), 1.93 (s, 2H), 1.88 (ddt, J = 3.9, 7.8, 11.3 Hz, 1H), 1.63 (ddd, J = 2.0, 4.1, 12.8 Hz, 2H), 1.36 (t, J = 6.5 Hz, 2H), 1.15 – 1.33 (m, 20H), 0.94 (s, 6H), 0.84 (m, 3H).

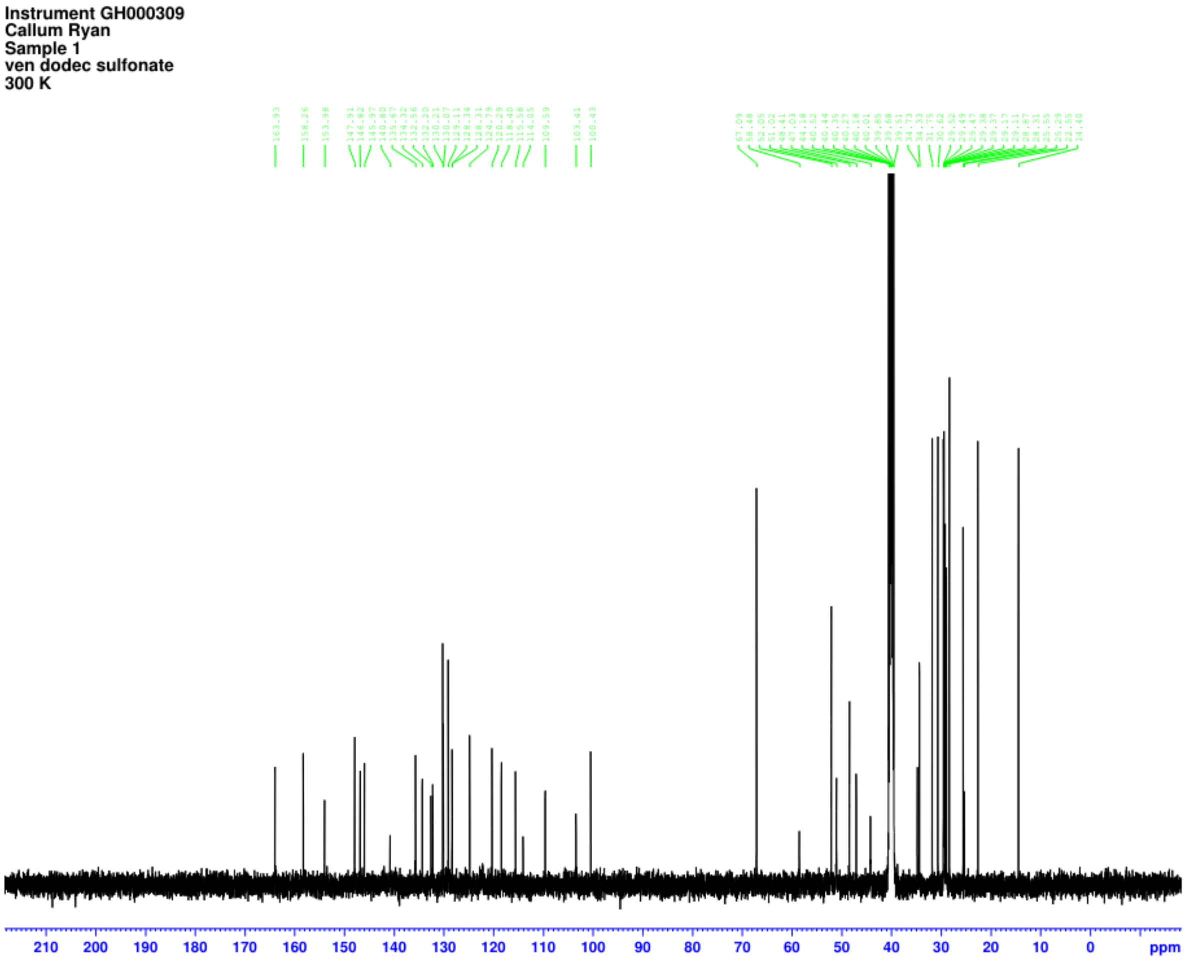

Figure S21: Venetoclax Dodecyl Sulfonate ^{13}C NMR in DMSO – d6 at 300K, 500 MHz: δ 163.93, 158.26,153.98, 147.91, 146.82, 145.97, 140.80, 135.67, 134.32, 132.56, 132.20, 130.21, 130.07, 129.11, 128.34, 128.31, 124.79, 120.29, 118.40, 115.58, 114.05, 109.59, 103.41, 100.43, 67.09, 58.48, 52.05, 51.02, 48.41, 47.03, 44.18, 40.52, 40.44, 40.35, 40.27, 40.18, 40.01, 39.85, 39.68, 39.51, 34.73, 31.75, 30.62, 29.52, 29.49, 29.47, 29.38, 29.37, 29.17, 29.11, 28.87, 28.31, 25.55, 25.29, 22.55, 14.40.

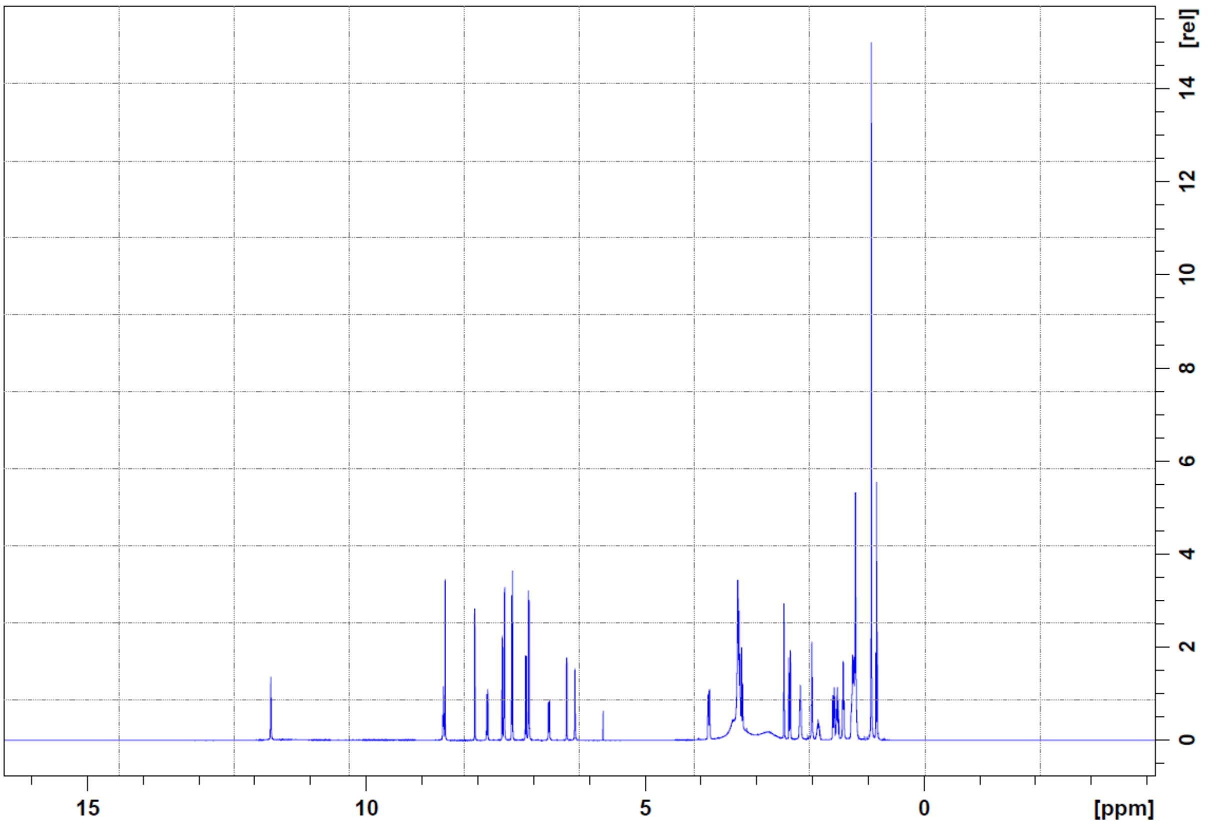

Figure S22: Venetoclax Octyl Sulfonate ^{1}H NMR in DMSO – d6 at 300K, 500 MHz δ 11.72 (m, 1H), 8.56 – 8.65 (m, 2H), 8.06 (d, J = 2.5 Hz, 1H), 7.84 (dd, J = 2.0, 8.8 Hz, 1H), 7.49 (d, J = 2.6 Hz, 1H), 7.47 – 7.53 (m, 2H), 7.27 – 7.36 (m, 2H), 7.12 (d, J = 9.4 Hz, 1H), 6.98 – 7.05 (m, 2H), 6.61 (dd, J = 2.3, 9.2 Hz, 1H), 6.39 (dd, J = 1.9, 3.4 Hz, 1H), 6.22 (d, J = 2.6 Hz, 1H), 3.89 (ddd, j = 2.0, 4.5, 11.4 Hz, 2H), 3.08 – 3.61 (m, 11H), 2.77 (s, 2H), 2.22 (d, J = 6.1 Hz, 4H), 2.15 (d, J = 6.5 Hz, 2H), 1.93 (s, 2H), 1.88 (ddt, J = 3.9, 7.8, 11.3 Hz, 1H), 1.63 (ddd, J = 2.0, 4.1, 12.8 Hz, 2H), 1.36 (t, J = 6.5 Hz, 2H), 1.15 – 1.33 (m, 14H), 0.94 (s, 6H), 0.84 (m, 3H).

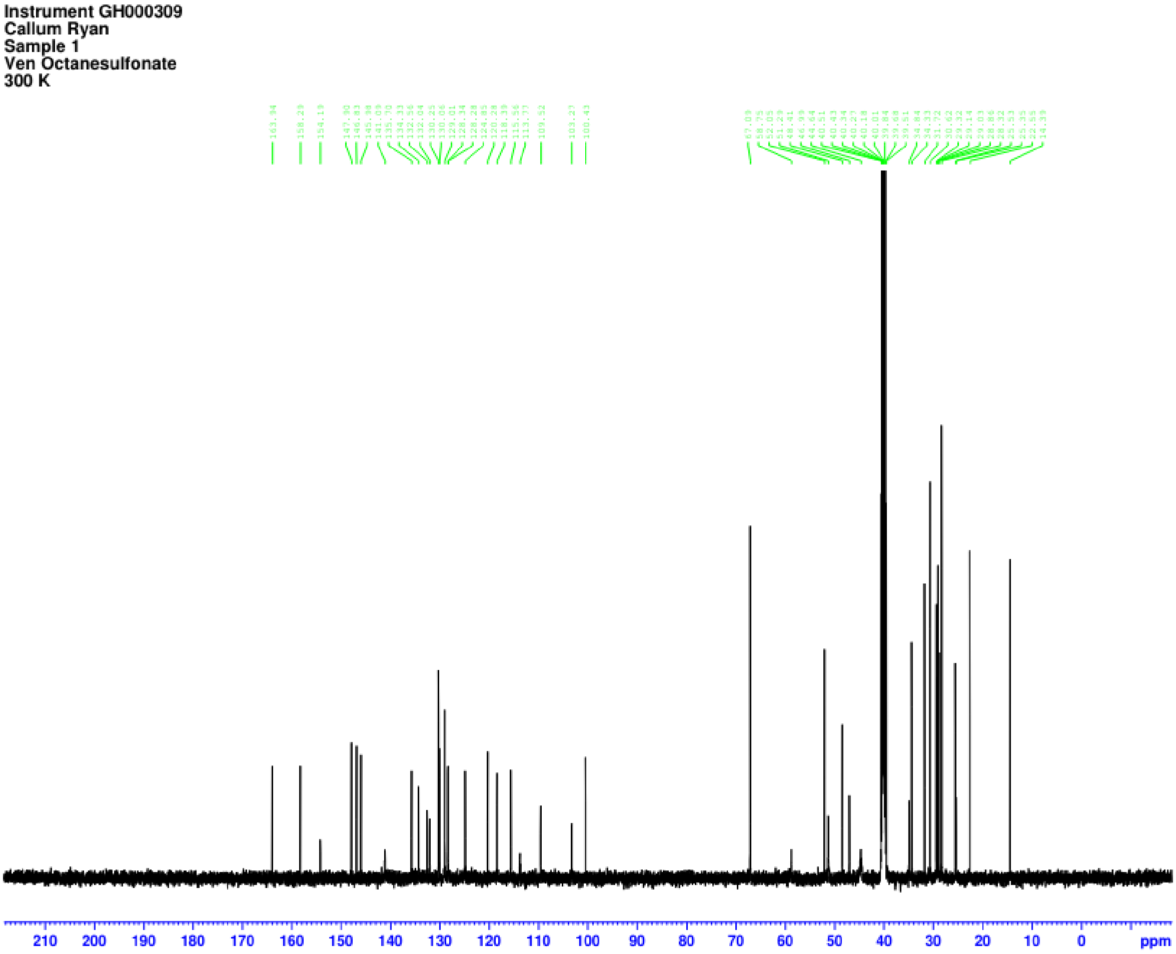

Figure S23: Venetoclax Octyl Sulfonate ^{13}C NMR in DMSO – d6 at 300K, 500 MHz: δ 163.94, 158.29, 154.20, 147.90, 146.83, 145.98, 141.11, 135.70, 134.33, 132.56, 132.03, 130.26, 130.07, 129.01, 128.34, 128.29, 124.84, 120.28, 118.39, 115.57, 113.80, 109.52, 103.27, 100.43, 67.09, 58.79, 52.06, 51.29, 48.41, 46.99, 44.65, 40.61, 40.51, 40.44, 40.35, 40.27, 40.18, 40.01, 39.85, 39.68, 39.51, 34.84, 34.33, 31.73, 30.62, 29.33, 29.15, 29.03, 28.87, 28.32, 25.55, 25.35, 22.55, 14.39

A

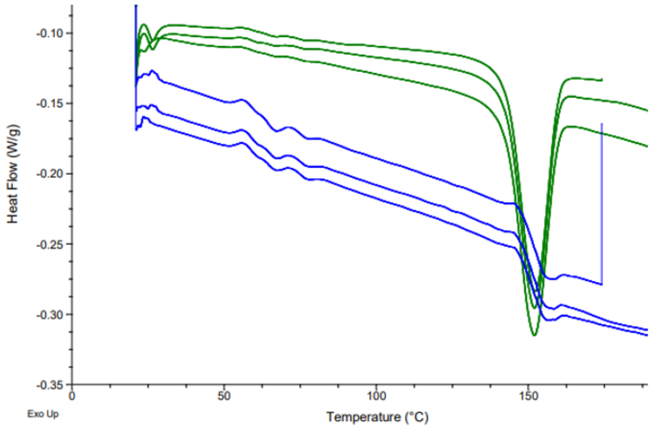

Figure S24: DSC plot for venetoclax docusate.

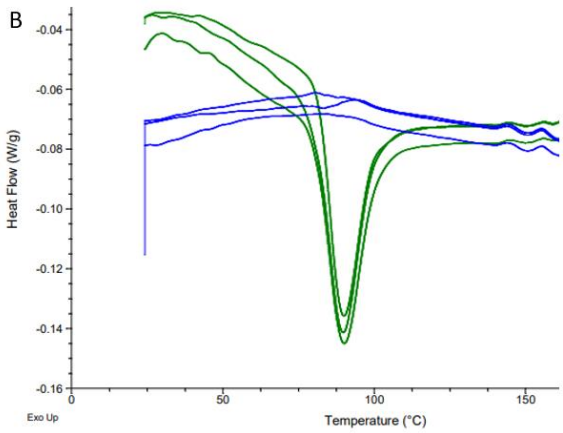

Figure S25: DSC plot for venetoclax octadecyl sulfate.

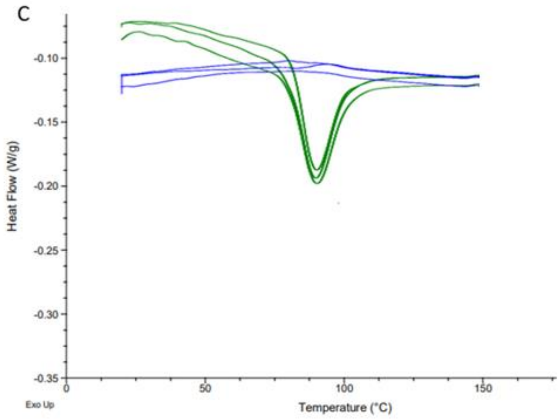

Figure S26: DSC plot for venetoclax dodecyl sulfate.

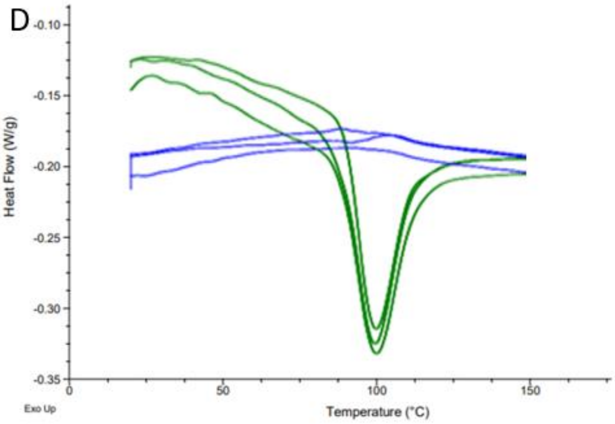

Figure S27: DSC plot for venetoclax decyl sulfate.

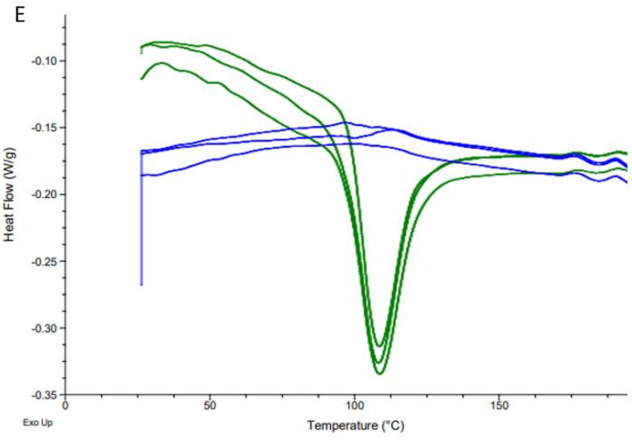

Figure S28: DSC plot for venetoclax octyl sulfate.

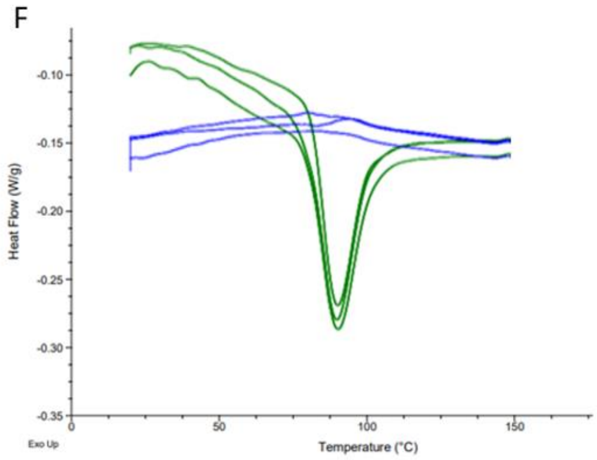

Figure S29: DSC plot for venetoclax dodecyl sulfonate.

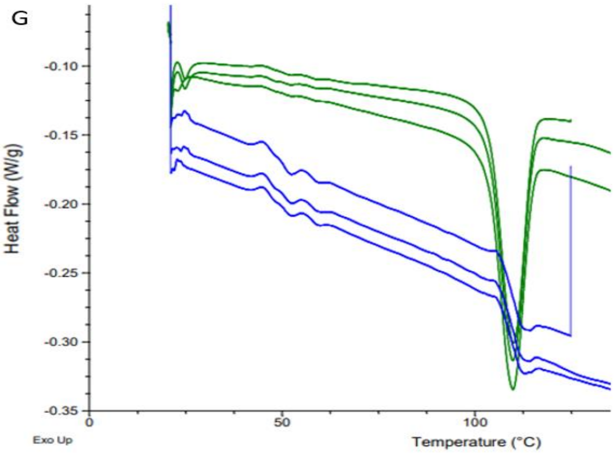

Figure S30: DSC plot for venetoclax octyl sulfonate.
